# Highly Efficient Removal of Neonicotinoid Insecticides by Thioether-Based (Multivariate) Metal–Organic Frameworks

**DOI:** 10.1021/acsami.1c08833

**Published:** 2021-06-14

**Authors:** Cristina Negro, Héctor Martínez Pérez-Cejuela, Ernesto F. Simó-Alfonso, José Manuel Herrero-Martínez, Rosaria Bruno, Donatella Armentano, Jesús Ferrando-Soria, Emilio Pardo

**Affiliations:** † Instituto de Ciencia Molecular (ICMol), Universidad de Valencia, 46980 Paterna, Valencia, Spain; ‡ Departamento de Química Analítica, Universitat de València, c/Dr. Moliner, 50, 46100 Burjassot, Valencia, Spain; § Dipartimento di Chimica e Tecnologie Chimiche (CTC), Università della Calabria, Rende 87036, Cosenza, Italy

**Keywords:** multivariate metal−organic frameworks, amino acids, water remediation, neonicotinoid insecticides, host−guest chemistry, crystal structures

## Abstract

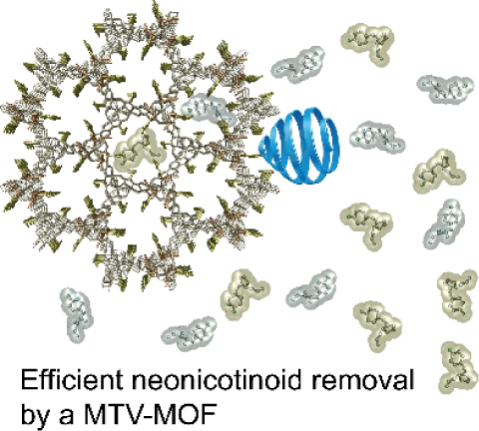

Circumventing the impact of agrochemicals on aquatic environments has become a necessity for health and ecological reasons. Herein, we report the use of a family of five eco-friendly water-stable isoreticular metal–organic frameworks (MOFs), prepared from amino acids, as adsorbents for the removal of neonicotinoid insecticides (thiamethoxam, clothianidin, imidacloprid, acetamiprid, and thiacloprid) from water. Among them, the three MOFs containing thioether-based residues show remarkable removal efficiency. In particular, the novel multivariate MOF {Sr^II^Cu^II^_6_[(*S,S*)-methox]_1.5_[(*S,S*)-Mecysmox]_1.50_(OH)_2_(H_2_O)}·36H_2_O (**5**), featuring narrow functional channels decorated with both −CH_2_SCH_3_ and −CH_2_CH_2_SCH_3_ thioalkyl chains—from l-methionine and l-methylcysteine amino acid-derived ligands, respectively—stands out and exhibits the higher removal efficiency, being capable to capture 100% of acetamiprid and thiacloprid in a single capture step under dynamic solid-phase extraction conditions—less than 30 s. Such unusual combination of outstanding efficiency, high stability in environmental conditions, and low-cost straightforward synthesis in **5** places this material among the most attractive adsorbents reported for the removal of this type of contaminants.

## Introduction

Neonicotinoids^1^ (NEOs)—so-called because of their chemical resemblance to nicotine alkaloid—are a widely used type of insecticides that, despite some recent restrictions,^2^ have extensively spread throughout the world in the past decades because of their high efficiency in controlling insect pests. However, their use is also associated with significant environmental concerns.^1^ Thus, despite low toxicity for beneficial insects was reported initially, subsequent studies demonstrated potential toxicity to beneficial insects^3^—such as honeybee colonies—as well as an alarming impact on avian species biodiversity, especially on grassland and insectivorous bird populations.^4^ In this context, another feature of NEO insecticides, which explain to a certain extent their popularity, is their moderate water solubility, which facilitates their application to soils and plant adsorptions.^1^ This point also constitutes a problem, from an environmental point of view, because of the concomitant contamination of aquatic environments.^5^ As a consequence, it is clear that as long as NEOs are not definitely banned, efficient capture technologies are needed.

Different technologies have been proposed for the removal/degradation of pesticides and insecticides.^6,7^ These include precipitation, coagulation/flocculation, membrane technologies, use of biological processes, advanced oxidation processes, or adsorption by porous sorbents.^5,8−10^ Among them, the removal of these contaminants by a porous material offers potential advantages over other technologies, such as, for example, preventing the formation of secondary contaminants and offering the possibility to implement economically viable decontamination protocols worldwide—which is of main relevance, especially in developing countries, giving a global application character to such potent technology.^11^

Metal–organic frameworks^12−16^ (MOFs) are porous crystalline materials that, among many other properties, have already demonstrated to be highly efficient in the removal of both organic and inorganic contaminants.^6,11,17−25^ Main reasons for such efficiency are high water and structural stability,^26,27^ microporosity that can be functionalized pre- or postsynthetically,^28^ to increase affinity for contaminants, and a certain degree of flexibility and/or adaptability that may play a key role capturing and accommodating the guest target contaminant.^29,30^ Moreover, unlike other porous materials, such thrilling host–guest chemistry can be visualized with the precious help of single-crystal X-ray diffraction^31,32^ (SCXRD), given the high crystallinity of these porous materials.^33,34^ This last point has been demonstrated to be extremely useful to unveil host–guest interaction governing the mechanism of the capture processes. More recently, a particular type of MOFs, the so-called multivariate MOFs^35−37^ (MTV-MOFs), which combine organic linkers with different functional groups decorating their channels, has emerged strongly in different fields that include water remediation.^11^ However, despite all these remarkable features, the use of MOFs for the sensing^38−41^ and/or removal of pesticides/insecticides has been only barely explored.^42−48^

**Figure 1:**
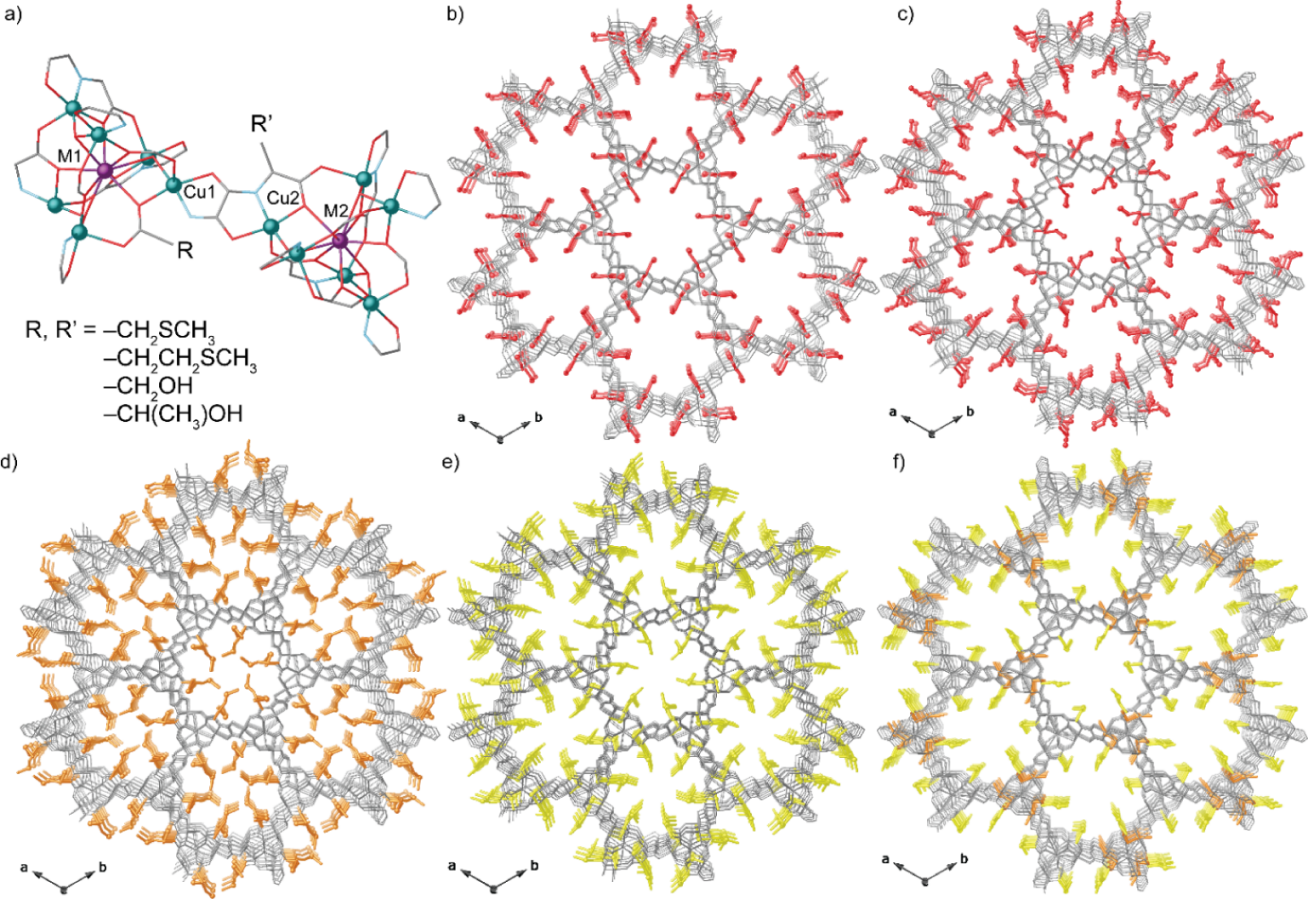
(a) Fragment of the structure of MOFs 1–5 emphasizing the common dicopper(II) building block. Copper and calcium/strontium (M) atoms from the network are represented by cyan and purple spheres, respectively, whereas organic ligands are depicted as gray (C), blue (N), and red (O) sticks. Perspective views of MOFs **1** (b), **2** (c), **3** (d), **4** (e), and **5** (f) along the *c* axes. Metals and organic ligands are depicted as gray sticks, whereas the amino acid residues are represented with the following color code: −CH_2_OH(1)/–CH(CH_3_)OH(2) (red), −CH_2_CH_2_SCH_3_ (**3** and **5**) (orange), and −CH_2_SCH_3_ (**4** and **5**) (yellow).

In this work, we explore the performance of a family of five water-stable highly crystalline three-dimensional (3D) isoreticular MOFs (one of them is MTV-MOFs) in the removal of different NEOs of environmental concern from water. In particular, we have focused on the use of four previously reported MOFs, with formulas {Ca^II^Cu^II^_6_[(*S,S*)-serimox]_3_(OH)_2_(H_2_O)}·39H_2_O^49^ (**1**), {Sr^II^Cu^II^_6_[(*S,S*)-threomox]_3_(OH)_2_(H_2_O)}·36H_2_O^50^ (**2**), {Ca^II^Cu^II^_6_[(*S,S*)-methox]_3_(OH)_2_(H_2_O)}·16H_2_O^29,30^ (**3**), and {Ca^II^Cu^II^_6_[(*S,S*)-Mecysmox]_3_(OH)_2_(H_2_O)}·16H_2_O^51,52^ (**4**) (where serimox = bis[(*S*)-serine]oxalyl diamide; threomox = bis[(*S*)-threonine]oxalyl diamide; methox = bis[(*S*)-methionine]oxalyl diamide; and Mecysmox = bis[*S*-methylcysteine]oxalyl diamide), and a novel MTV-MOF of formula {Sr^II^Cu^II^_6_[(*S,S*)-methox]_1.5_[(*S,S*)-Mecysmox]_1.50_(OH)_2_(H_2_O)}·36H_2_O (**5**) (Scheme S1 and Figure 1). The selection of this family of MOFs is not accidental. This is based on their good mechanical properties, which have already permitted their processability as pellets^29^ or mixed matrix membranes,^51^ their proven air and water stability^53^ (neutral and basic media), and their excellent performances in the removal of both inorganic^29,51^ (Hg^2+^) and organic contaminants (dyes^54^ or vitamins,^49,55^ recently reported for **1**–**4**, in the presence of other metal cations and inorganic anions usually present in potable drinking water.^29,30,35,54^ The main reasons that lie at the origin of such remarkable capture properties are twofold: (i) these MOFs feature channels decorated with different functional groups, which can be tuned depending on the nature of the chosen amino acid residue (−CH_2_OH, −CH(CH_3_)OH, −CH_2_SCH_3_, and/or −CH_2_CH_2_SCH_3_); and (ii) these amino acid residues exhibit a high degree of adaptability,^56^ being capable of accommodating and adjusting to guest molecules by maximizing host–guest interactions with the contaminant.

## Results and Discussion

We report here the efficiency of the whole family of MOFs, as solid-phase extraction (SPE) sorbents, toward five well-known NEOs like thiamethoxam, clothianidin, imidacloprid, acetamiprid, and thiacloprid (Scheme S2 and Table S1).^1^ Overall, the three thioether-derived isoreticular MOFs (**3**–**5**) are capable of capturing, very efficiently, NEOs in a single loading step—within 30 s. In particular, the novel MTV-MOF **5**—featuring functional pores tailored with, approximately, a 50% of −CH_2_CH_2_SCH_3_ groups and another 50% of the −CH_2_SCH_3_ residues from the amino acids l-methionine and *S*-methyl-l-cysteine (Figure 1f), respectively—shows the best performance for all the NEOs, being capable of capturing 99–100% of acetamiprid and thiacloprid in a single capture process. In addition, it has been possible to solve the crystal structures of the resulting host–guest adsorbates with acetamiprid and thiacloprid that help to unveil the mechanism of the capture process of MTV-MOF **5**.

Figure 1 shows the crystal structures of **1**–**5**. They all are isomorphous, crystallizing in the chiral *P*6_3_ space group of the hexagonal system, and exhibit chiral 3D calcium(II)/strontium(II)–copper(II) networks featuring hexagonal channels, where the different adaptable amino acid residues are depicted in different colors (see color code in Figure 1). The crystal structure of the novel material reported here {Sr^II^Cu^II^_6_[(*S,S*)-methox]_1.5_[(*S,S*)-Mecysmox]_1.50_(OH)_2_(H_2_O)}·36H_2_O (**5**) has been determined by SCXRD measurements—using synchrotron radiation at the I19 beamline of the Diamond Light Source (Table S2). Well-shaped crystals of **5** were grown with a slow diffusion technique (see the Supporting Information). **5** presents an uninodal **acs** six-connected 3D strontium(II)–copper(II) network with functional hexagonal channels—virtual diameters of ca. 1 nm—decorated by the two types of flexible amino acid residues, the ethylene- (−CH_2_CH_2_SCH_3_) and the methylene-thiomethyl (−CH_2_SCH_3_), belonging to methionine and methyl cysteine amino acids, respectively (Figures 1 and S1).

Similarly to single-ligand parent compounds **3** and **4**, MTV-MOF **5** shows also a highly stable 3D porous network, with flexibility confined only in pores, where highly bendable arms are prone to adopt different conformations of the thioether chains depending on the different chemical environments determined by guest’ s nature (Figure S1c,d). In particular, the crystal structure of **5** shows methionine arms more bent than methyl-cysteine ones (Figures 1 and S1a,b), featuring available sulfur groups for interaction—mainly based on σ-hole–with electron donors—including oxygen and nitrogen atoms^57−61^ and π-systems (vide infra).^62^ Thus, they encapsulate the targeted guest molecules assuming the favorite conformation, in each case, to maximize the host–guest interactions. The channel size in **5** (Figure S1b) is similar to those in **3** and **4**, with the added value of chemical diversity confined in the same pores, guaranteed by diverse length and electron density for −CH_2_CH_2_SCH_3_ and −CH_2_SCH_3_ groups. The crystal structure of the chiral network **5** unveils statistically disordered *trans*-oxamidato-bridged dicopper(II) units of {Cu^II^_2_[(S,S)-Mecysmox]} and {Cu^II^_2_[(S,S)-methox]} (Figure S1 inset,c), which build the 3D motif. As stated above, SCXRD measurements on **5** have been performed using synchrotron radiation. This was done with the aim to safeguard the desirable high quality of data set in case of such statistical disordered. In this respect, the best final model found for the crystal structure is based on the most realistic assumption that there is a random distribution of methyl-cysteine and methionine moieties (with 1:1 ratio) within the net (see crystallographic details in the Supporting Information), as previously reported by us for an analogue MTV-MOF.^35^ In so doing, the spatial average, of all fragments and all their possible orientations averaged in the crystal via only one unit cell (see crystallographic details in the Supporting Information), discloses basically the crystal structure of **5**, which is constructed from the self-assembly of copper(II) dimers and Sr^II^ ions, through the carboxylate groups of the ligands (Figure S1). Aqua/hydroxo groups (in a 1:2 statistical distribution) contribute to further connect neighboring Cu^2+^ and Cu^2+^/Sr^2+^ ions finally linked in a μ_3_ fashion (Figures S1c,d). Indeed, it must be the comparable percentage of Mecysmox and methox that gives back to superimposed snapshot of mixed {Cu^II^_2_[(*S*,*S*)-methox/Mecysmox]} dimers, which is also supported by the experimental results of composition analysis (vide infra C, H, S, N, and Supporting Information).

Besides the structural characterization and elemental analysis, the chemical identity of **5** was further stablished by powder X-ray diffraction (PXRD), electronic microscopy, and thermogravimetric analyses (TGAs) (see the Supporting Information).

Figure S2 shows the experimental PXRD pattern of **5**. It is identical to the theoretical one, which confirms that the bulk sample is pure and homogeneous. Moreover, the structural stability of **5** was tested after being soaked, for 48 h, in neutral (Figure S3b), basic (Figure S3c, pH = 12), and acid (Figure S3d, pH = 5 and Figure S3e, pH = 2) aqueous media (Figure S3). This test confirmed that **5** is stable in basic and moderately acid media. The permanent porosity of **5** was verified by measuring their N_2_ adsorption isotherm at 77 K, which is also compared to those adsorption isotherms of related MOFs **3** and **4** (Figure S4). Overall, they confirm permanent porosity for **3**–**5**, with larger N2 adsorbed amounts for **4** and **5**, which is consistent with higher accessible void spaces, as suggested by the crystal structures (Figure 1). The solvent content of **5** was, however, definitively established by TGA (see Figure S5), which also confirms that **5** is stable up to 250 °C, when decomposition starts. The reported analyses performed both on the bulk and on the crystal sample of MTV-MOF **5** unveil similar composition to that used in the reaction mixture, validating the hypothesis that there were no significant ligand preferences giving nature and stability of both **3** and **4** parent MOFs. These results, together with previously reported ones,^35^ confirm a successful protocol, which proposes that the composition can be controlled through the relative reactant concentrations in this family of materials.

For the evaluation of the NEO capture properties, SPE devices were prepared by packing 25 mg of the corresponding MOF (**1**–**5**) between two frits into 1 mL of empty propylene cartridges. First of all, activation and equilibration of the sorbent were done with 1 mL of MeOH and 1 mL of H_2_O, consecutively. Then, 1 mL of aqueous mixtures of thiamethoxam, clothianidin, imidacloprid, acetamiprid, and thiacloprid at four levels of concentrations (0.1, 1, 10, and 100 mg L^–1^) was percolated through the SPE cartridges. Then, a washing step was carried out with 1 mL of H_2_O. After that, elution of the retained analytes was accomplished with MeOH (5 mL). All SPE fractions were collected and filtered (membrane with pore size of 0.22 μm) prior to their injection in HPLC system, and the NEO content was established (see also Experimental Section).

Following this procedure, the efficiency of the NEO capture was, initially, evaluated for **1**–**5** using a mixture of the five NEOs (at 1 mg L^–1^ each), and the results are collected in Table S3. Overall, the five MOFs showed distinct behaviors, and they can be classified in two clearly distinct groups. The serine- (**1**) and threonine-derived (**2**) MOFs—for which a high capture efficiency of organic dyes was reported previously—exhibit, by far, much worse capture properties than the thioalkyl MOFs (**3**–**5**) (Table S3). **1** and **2** do not surpass 20% of removal efficiency for any of the NEOs. In turn, **3**, **4**, and, especially, the MTV-MOF **5** show much higher efficiencies. On this basis, MOFs **3**–**5** were subjected to further capture experiments using different contents of the five NEOs (0.1, 10, and 100 mg L^–1^). In so doing, it was observed that, overall, the three MOFs capture, very efficiently, thiacloprid and acetamiprid and, moderately well, clothianidin, imidacloprid, and thiamethoxam (Table 1). In particular, MTV-MOF **5** exhibits outstanding capture properties, especially at very diluted conditions. Thus, **5** captures, in a single step, 100% of thiacloprid and acetamiprid in any condition and 71–86%, at the most diluted conditions, of clothianidin, imidacloprid, and thiamethoxam.

**Table 1:** Removal Values (%) for NEOs from Different Aqueous Samples (at Three Levels of Concentration) Using MOFs 3–5 (*n* = 3)

		MOF		
NEOs	concentration (mg L^–1^)	3	4	5
thiamethoxam	0.1	66	45	71
	10	30	28	33
	100	33	25	30
clothianidin	0.1	60	48	86
	10	64	48	74
	100	47	43	61
imidacloprid	0.1	65	50	86
	10	50	42	57
	100	38	41	60
acetamiprid	0.1	95	91	99
	10	96	91	99
	100	86	94	100
thiacloprid	0.1	93	96	100
	10	91	96	100
	100	87	98	100

To further confirm the applicability of the developed method in removing NEOs from environmental matrices with possible competing species, a real water samples from river (Turia river; 39.504095, −0.473712; Valencia) were analyzed. For real sample analysis (river water), the same SPE protocol described above was used. None of the target pollutants were found in the samples using the optimized protocol (see Experimental Section). Therefore, the river water was spiked at 5 mg L^–1^ with each of the five NEOs. As it can be seen in Figure S6, a significant decrease in the signal was observed after SPE treatment with MOFs **3–5** as sorbents, indicating the suitable removal efficiency of these MOFs for organic pollutants in environmental waters (Table S4). Furthermore, the reproducibility of these sorbents was evaluated, as relative standard deviation, showing values lower than 9% for all the analytes (Table S5).

**Figure 2:**
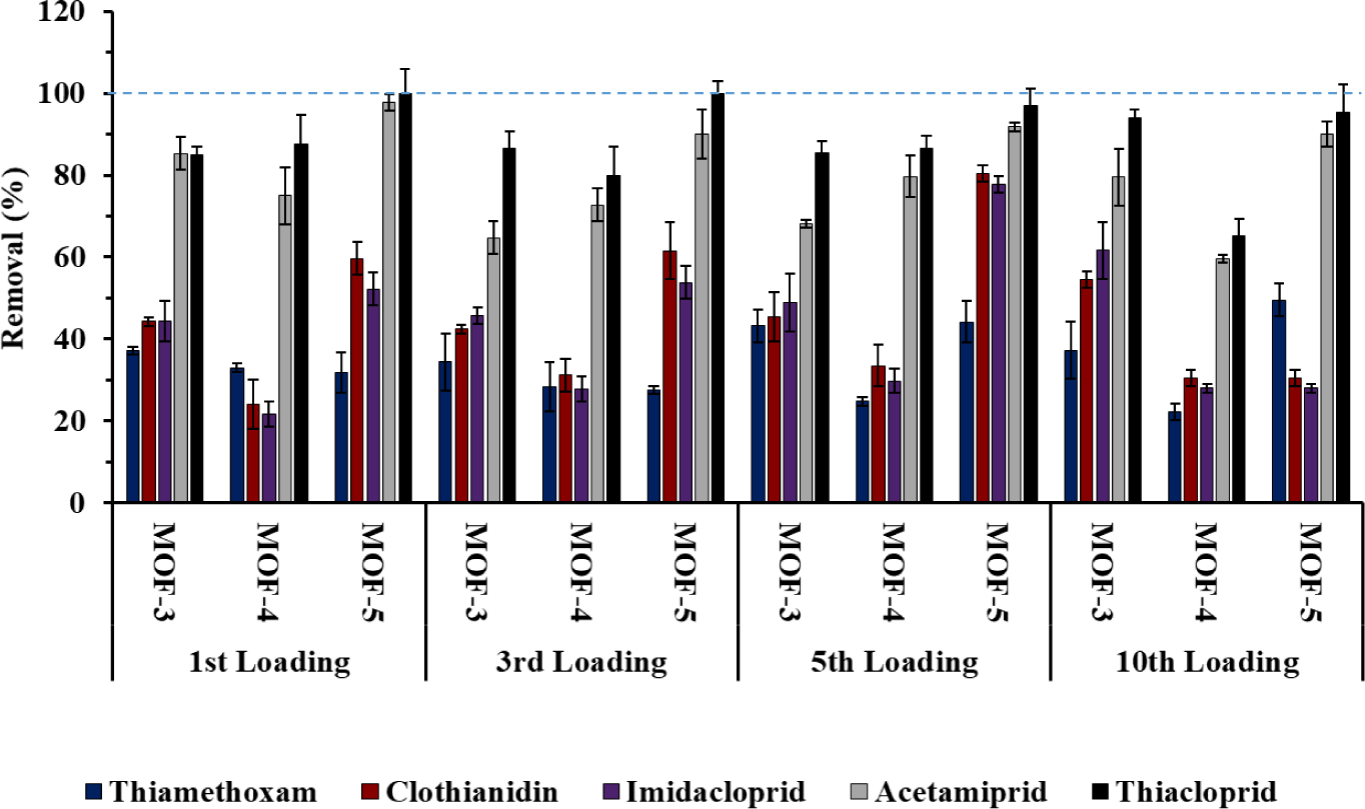
Reuses of **3**–**5** for the removal (%) of thiamethoxam, clothianidin, imidacloprid, acetamiprid, and thiacloprid using a 1 mg L^–1^ mixture of NEOs.

In order to evaluate the reusability of the MOFs, up to 10 capture cycles—using a mixture of the five NEOs (at 1 mg L^–1^ level)—were performed for **3**–**5** (Figure 2). For this purpose, the same SPE-optimized protocol, used before (see Experimental Section), was followed using an aqueous standard mixture of the five NEOs, at 1 μg mL^–1^. It can be observed that, at least for 10 cycles, the three MOFs maintain the efficiency for the removal of the five NEOs, showing a similar capture performance. Figure S7 shows the PXRD patterns, after 10 consecutive NEO sorption/desorption cycles, which confirm that **3**–**5** maintain the structural integrity. Moreover, no metal leaching could be observed in any of the sorption/desorption cycles.

Finally, we evaluated the maximum loading capacity of the best performing materials (**3**–**5**) toward each of the selected NEOs. Thus, polycrystalline samples of **3**–**5** were soaked in saturated water/acetonitrile (1:1) solutions of thiamethoxam, clothianidin, imidacloprid, acetamiprid, and thiacloprid for 1 week, replacing each saturated solution every 24 h (see Experimental Section). In so doing, maximum uptakes of 275, 312, 356, 426, and 411 (**3**); 402, 321, 399, 423, and 415 (**4**); and 447, 379, 402, 445, and 499 (**5**) mg g^–1^ were determined for thiamethoxam, clothianidin, imidacloprid, acetamiprid, and thiacloprid, respectively. Maximum loadings observed for **5** closely corresponds to up to three guest molecules per SrCu_6_ formula unit.

**Figure 3:**
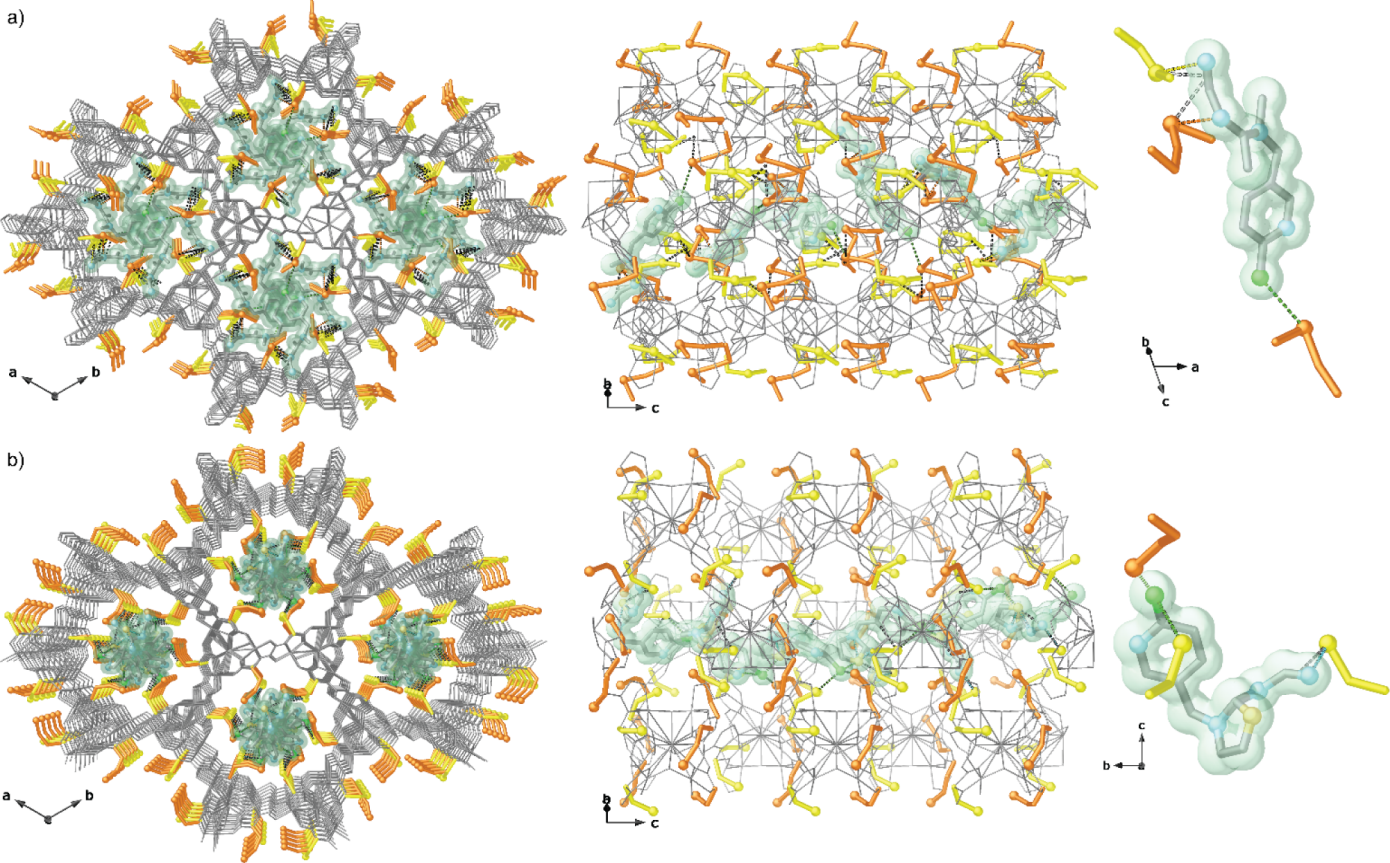
Perspective views in the *ab* (left) and *bc* (middle) planes of the porous structures of **acetamiprid@5** (a) and **thiacloprid@5** (b). Metals and organic ligands from the network are represented as in Figure 1, whereas the guest NEO molecules are represented as light blue (nitrogen), green (chloride), pale yellow (sulfur) ball-and-stick, and gray (carbon) sticks. Guest molecules are also represented as green solid surfaces with the same color code for atoms. The guest molecule structures are shown in detail in the right side of the porous structures.

On the basis of these results and aiming at elucidating the mechanisms involved in the capture processes of the best performing material, insertion experiments were also carried out on single crystals of MTV-MOF **5 (**see the Supporting Information and Experimental Section). Remarkably, suitable samples of host–guest aggregates of **5** with acetamiprid and thiacloprid for SCXRD were obtained, and the crystal structure of **acetamiprid@5** and **thiacloprid@5** could be determined (Table S2), which allowed the atomically precise visualization on the interaction of the two most efficiently captured NEO pollutants with the thioalkyl residues decorating the framework. The chemical formulas were finally established with the help of CHNS and SEM/EDX analyses (see Experimental Section and the Supporting Information), and the solvent contents were estimated by TGA: acetamiprid@{Sr^II^Cu^II^_6_[(*S,S*)-methox]_1.5_[(*S,S*)-Mecysmox]_1.50_(OH)_2_(H_2_O)}·9H_2_O (**acetamiprid@5**) and thiacloprid@{Sr^II^Cu^II^_6_[(*S,S*)-methox]_1.5_[(*S,S*)-Mecysmox]_1.50_(OH)_2_(H_2_O)}·18H_2_O (**thiacloprid@5**) (Figures 3 and S5).

Compounds **acetamiprid@5** and **thiacloprid@5** are isomorphous to **5** and crystallize in the *P*6_3_ chiral space group of the hexagonal system, confirming the preservation of the 3D network of the hosting matrix **5** even after the guests’ capture. The crystal structures clearly evidence that acetamiprid and thiacloprid guest molecules are encapsulated in the nanopores of **5**, where they are simultaneously recognized by the thioether arms of the methyl-cysteine and methionine residues. The most stabilizing forces are assured by sulfur atoms interacting either with nitrile groups or with Cl atoms as electron donors. Although the different chemical nature of acetamiprid and thiacloprid**—**featuring nitrile groups—among the whole family of tested NEOs, seems a priori to be discriminant, it is not supported by host–guest interactions visualized by SCXRD. Indeed, it is pretty interesting to observe that in **acetamiprid@5** both methyl-cysteine and methionine arms distend their conformation within pores pointing toward nitrile groups, while in **thiacloprid@5**, the S···Cl interaction is observed as prominent—with molecules orienting in such a way to confine the −CN moieties toward the hidden center of the pores (Figures 2 and S8–S11). Although both acetamiprid and thiacloprid molecules were disordered in the pores, we succeeded to get their possible configurations and locations (see Supporting Information for structural details) as well as details on their interaction sites with the hosting matrix MTV-MOF **5** (Figures S8–S13).

Details of **acetamiprid@5** crystal structure show molecules statistically disordered on three configuration sets (see Figures S8 and S12) residing in the pores, packed via straight S···N–CN—involving only methionine residues—[S···N distances of 3.18(1) Å] and S···nitrile interactions—involving both kind of arms—which block acetamiprid terminal moieties at almost identical distance [S···CN_Centroid_ distances of 3.67(1) and 3.82(1) Å, for methyl-cysteine and methionine residues, respectively] (Figure S9). On the contrary, in **thiacloprid@5** crystal structure (Figure S10), the two kinds of amino acid residues are involved in different contacts. Thiacloprid molecules, statistically disordered as well on three configuration sets (Figure S13), are captured via either methionine residues, which involve sulfur atoms to interact with Cl [S···Cl distance of 3.10 (1) Å] or methyl-cysteine residues, which contribute with interactions of the type S···S held with thiazolidine ring of pollutant molecules [S···S distance of 2.76(1) Å] (Figure S11). Both contact distances fall in the range of those found in the literature for similar S interactions,^61^ although the last distance, exhibiting a value lower than the sum of van der Waals radii, has been rarely observed.^63^ The arrangement of the NEO molecules is clearly driven by the pore’s size as well, which imposes preferential configurations. Indeed, despite their structural similarity, the molecular orientation found in the nanoconfined space for acetamiprid and thiacloprid is surprisingly different. It is worth to note also the high loading of guests, which displace almost all water molecules from the pores, and it is at the origin of the close-packing observed. In fact, **acetamiprid@5** and **thiacloprid@5** almost totally fill channels (Figures S8 and S10), making extremely robust the adsorbates being stable at air and room temperature for 4 weeks. This, indeed, represents an added value for a more safe storage and handling of a scavenger material like that, for which only the regeneration process, based on the use of appropriate solvent, will cause the release of captured pollutants.

The high performance of **5** for some NEO capture could be understood with the help of X-ray crystallography. Interactions found in **acetamiprid@5** and **thiacloprid@5**, discussed in the context of both sulfur-containing ligands, have a prominent role because of their extensive propagation in the nanoconfined space ensured by **5**, exactly as observed for peptide-based methionine, cysteine, and cysteine moieties—where associations extend beyond that of simple hydrophobic interactions. Both intra- and intermolecular interactions—involving low-lying sulfur σ* orbitals—are known to be implicated in chemical reactivity, with electronic characteristics of chemical systems responsible, in part, for specific kinetic, regiochemical, or even stereochemical outcomes. Indeed, it is also known that electron-deficient bivalent sulfur atoms have two areas of positive electrostatic potential, as a consequence of the low-lying σ* orbitals of the C–S bond (the so-called σ-hole),^62,64^ which are available for interaction with electron donors such as nitrogen atoms or, as in the present case, nitrile groups and, even, π-systems. The present results, together with the previously reported by us,^20,25−27^ represent the first examples of a judicious exploitation of these sulfur-based interactions. Intramolecular interactions are by far the most common manifestation of this effect, which offers a means of modulating the conformational preferences of a molecule. Although it is a well-documented phenomenon, a priori applications in rational capture are relatively sparse, and this interaction, which is often isosteric with an intramolecular hydrogen-bonding interaction, appears to be underappreciated by the applied chemistry community. The majority of the examples of this kind of sulfur interaction have been noted in post facto analyses of crystallographic or other structural information, and there are relatively few examples reported in the literature where this interaction has been exploited in a prospective fashion.^65^

## Conclusions

In summary, we report the one-step efficient capture of NEO insecticides by a family of isoreticular thioether-based MOFs derived from amino acids l-methionine and *S*-methyl-l-cysteine. In particular, the novel MTV-MOF **5**—combining both amino acids in equal proportions—exhibits outstanding capture properties, being capable to remove, in a single step, 100% of acetamiprid and thiacloprid at different conditions and 71–86%, at the most diluted conditions, of clothianidin, imidacloprid, and thiamethoxam. In addition, the capture properties are maintained during, at least 10 cycles. Remarkably, the crystal structures of the two host–guest aggregates of **5** with acetamiprid and thiacloprid could be resolved, which allowed to visualize how both NEOs are encapsulated and immobilized. Also, it enables to unveil the synergistic interactions of both types of thioether groups with the guest molecules, which are ultimately responsible for such capture efficiency. This family of thioether-containing MOFs arise as an alternative for more traditional materials, such as activated carbons,^66−68^ for the capture of this type of emerging contaminants.

## Experimental Section

### Preparation of {Sr^II^Cu^II^_6_[(*S,S*)-methox]_1.5_[(*S,S*)-Mecysmox]_1.50_(OH)_2_(H_2_O)}·36H_2_O (5)

Well-shaped hexagonal prisms of **5**, suitable for SCXRD, were obtained by slow diffusion in H-shaped tubes of aqueous solutions containing stoichiometric amounts of (Me_4_N)_2_{Cu_2_[(S,S)-methox](OH)_2_}·4H_2_O (0.131 g, 0.18 mmol) and (Me_4_N)_2_{Cu_2_[(S,S)-Mecysmox](OH)_2_}·5H_2_O (0.129 g, 0.18 mmol) in one arm and Sr(NO_3_)_2_ (0.025 g, 0.12 mmol) in the other. They were isolated by filtration on paper and air-dried. A gram-scale procedure was also carried out successfully by mixing greater amounts of (Me_4_N)_2_{Cu_2_[(S,S)-methox](OH)_2_}·4H_2_O (4.37 g, 6 mmol) and (Me_4_N)_2_{Cu_2_[(S,S)-Mecysmox](OH)_2_}·5H_2_O (4.32 g, 6 mmol) in water (60 mL). Another aqueous solution of Sr(NO_3_)_2_ (0.846 g, 4 mmol) was added dropwise to the resulting deep green solution, and the final mix was allowed to react, under stirring, for 6 h. Afterward, the material was isolated by filtration and characterized by C, H, N, S analyses to give a final formula of {Sr^II^Cu^II^_6_[(*S,S*)-methox]_1.5_[(*S,S*)-Mecysmox]_1.50_(OH)_2_(H_2_O)}·30H_2_O. Anal. Calcd for **5**: C_33_Cu_6_SrS_6_H_118_N_6_O_57_ (2172.6): C, 18.24; H, 5.47; S, 8.86; N, 3.87%. Found: C, 18.13; H, 5.52; S, 8.82; N, 3.93%; IR (KBr): ν = 1611 and 1606 cm^–1^ (C=O).

### Preparation of Acetamiprid@{Sr^II^Cu^II^_6_[(*S,S*)-methox]_1.5_[(*S,S*)-Mecysmox]_1.50_(OH)_2_(H_2_O)}·9H_2_O (Acetamiprid@5) and Thiacloprid@{Sr^II^Cu^II^_6_[(*S,S*)-methox]_1.5_[(*S,S*)-Mecysmox]_1.50_(OH)_2_(H_2_O)}·18H_2_O (Thiacloprid@5)

Well-shaped hexagonal prisms of **acetamiprid@5** and **thiacloprid@5**, suitable for SCXRD, could be obtained by soaking crystals of **5** (ca. 5.0 mg) for a week in saturated acetonitrile solutions containing acetamiprid and thiacloprid (recharging fresh saturated solutions daily). After this period, they were isolated by filtration, air-dried, and characterized by SCXRD, C, H, N, S, and TGA analyses to give, as final formulas, acetamiprid@{Sr^II^Cu^II^_6_[(*S,S*)-methox]_1.5_[(*S,S*)-Mecysmox]_1.50_(OH)_2_(H_2_O)}·9H_2_O (**acetamiprid@5**) and thiacloprid@{Sr^II^Cu^II^_6_[(*S,S*)-methox]_1.5_[(*S,S*)-Mecysmox]_1.50_(OH)_2_(H_2_O)}·18H_2_O (**thiacloprid@5**). The same synthetic was carried out, with identical results, with larger amounts of polycrystalline samples. Anal. Calcd for **acetamiprid@5**: C_43_Cu_6_ClSrS_6_H_75_N_10_O_30_ (1908.9): C, 27.06; H, 3.96; S, 10.08; N, 7.34%. Found: C, 27.09; H, 3.77; S, 10.02; N, 7.38%; IR (KBr): ν = 2233 (C≡N), 1641 (C=N), and 1611 and 1606 cm^–1^ (C=O). Anal. Calcd for **thiacloprid@5**: C_43_Cu_6_ClSrS_7_H_91_N_10_O_39_ (2101.0): C, 24.58; H, 4.37; S, 10.68; N, 6.66%. Found: C, 24.59; H, 4.31; S, 10.59; N, 6.69%; IR (KBr): ν = 2238 (C≡N), 1645 (C=N), and 16 011 and 1609 cm^–1^ (C=O).

Well-shaped hexagonal prisms of **acetamiprid@5** and **thiacloprid@5**, suitable for SCXRD, could be obtained by soaking crystals of **5** (ca. 5.0 mg) for a week in saturated acetonitrile solutions containing acetamiprid and thiacloprid (recharging fresh saturated solutions daily). After this period, they were isolated by filtration, air-dried, and characterized by SCXRD, C, H, N, S, and TGA analyses to give, as final formulas, acetamiprid@{Sr^II^Cu^II^_6_[(*S,S*)-methox]_1.5_[(*S,S*)-Mecysmox]_1.50_(OH)_2_(H_2_O)}·9H_2_O (**acetamiprid@5**) and thiacloprid@{Sr^II^Cu^II^_6_[(*S,S*)-methox]_1.5_[(*S,S*)-Mecysmox]_1.50_(OH)_2_(H_2_O)}·18H_2_O (**thiacloprid@5**). The same synthetic was carried out, with identical results, with larger amounts of polycrystalline samples. Anal. Calcd for **acetamiprid@5**: C_43_Cu_6_ClSrS_6_H_75_N_10_O_30_ (1908.9): C, 27.06; H, 3.96; S, 10.08; N, 7.34%. Found: C, 27.09; H, 3.77; S, 10.02; N, 7.38%; IR (KBr): ν = 2233 (C≡N), 1641 (C=N), and 1611 and 1606 cm^–1^ (C=O). Anal. Calcd for **thiacloprid@5**: C_43_Cu_6_ClSrS_7_H_91_N_10_O_39_ (2101.0): C, 24.58; H, 4.37; S, 10.68; N, 6.66%. Found: C, 24.59; H, 4.31; S, 10.59; N, 6.69%; IR (KBr): ν = 2238 (C≡N), 1645 (C=N), and 16 011 and 1609 cm^–1^ (C=O).

### Capture Experiments

Selected NEOs (thiamethoxam, clothianidin, imidacloprid, acetamiprid, and thiacloprid) in this study were obtained from Sigma-Aldrich (St. Louis, MO, USA). See details and structures in Table S1. All the solvents (e.g., methanol (MeOH) or acetonitrile (MeCN) and others) were of HPLC grade and purchased from VWR International Eurolab (Barcelona, Spain). Nanopure water was purified in Crystal B30 EDI Adrona deionizer (Riga, Latvia). Other nonspecific reagents were of analytical grade unless otherwise stated. SPE propylene cartridges of 1 mL (internal volume) and their respective frits (1/16′, 20 μm) were provided from Análisis Vínicos (Tomelloso, Spain). Individual standard solutions (at 1000 mg L^–1^) of NEOs were prepared in fresh MeOH and kept until their use at 4 °C. For daily work, standard mixtures were prepared by dilution from the stock solutions. Water samples from Turia river (39.504095, −0.473712; Valencia) were collected in dark glass bottles and stored at 4 °C until analysis. For real sample analysis (river water), the same SPE protocol was done. River water samples were spiked after reaching room temperature, and no more additional pretreatment steps were needed.

### General SPE Protocol

SPE cartridges were prepared as stated above. First, 1 mL of aqueous standard mixtures of contaminants, at the appropriate concentration, was loaded to the cartridge. Then, a washing step was carried out using 1 mL of water. After that, the elution was accomplished with 5 mL of pure MeOH. An additional step was performed for reconditioning the extraction unit passing through the cartridge 1 mL of MeOH and 1 mL of water. The process was repeated until a significant signal decrease was achieved. All the fractions were filtered (using a nylon membrane, 0.23 μm) previous to their injection to the HPLC-UV system.

### Maximum Loading Experiments

The maximum loading capacities of MOFs **3**–**5** toward each of the selected NEOs were determined by soaking polycrystalline samples of **3**–**5**, in saturated water/acetonitrile (1:1) solutions of thiamethoxam, clothianidin, imidacloprid, acetamiprid, and thiacloprid for 1 week. Each saturated solution was replaced every 24 h. After 1 week, polycrystalline samples were filtered, and the number of guest molecules was estimated by determining the Cu_6_Sr/Cl ratio with ICP–MS analyses (data not shown).

### X-ray Crystallographic Data Collection and Structure Refinement

Crystals of **5**, **acetamiprid@5**, and **thiacloprid@5** were selected and mounted on a MITIGEN holder in Paratone oil, and then quickly placed in a nitrogen stream cooled at 100 K to extract the best data set avoiding the possible degradation upon desolvation or exposure to air. Nevertheless, crystals of both **acetamiprid@5** and **thiacloprid@5** samples displayed an outstanding stability at air and room temperature for at least 4 weeks, as demonstrated by their diffraction patterns measured at 296 K as well, without displaying any important crystal decay. Diffraction data for **5** were collected using synchrotron radiation at I19 beamline of the Diamond Light Source at λ = 0.6889 Å, whereas for **acetamiprid@5** and **thiacloprid@5** data were acquired on a Bruker-Nonius X8APEXII CCD area detector diffractometer using graphite-monochromated Mo Kα radiation (λ = 0.71073 Å), as a significant beam damage was observed for both single crystals under synchrotron radiation. Further crystallographic details can be found in the Supporting Information.

### X-ray Powder Diffraction Measurements

Fresh polycrystalline samples of **5**, **acetamiprid@5**, and **thiacloprid@5** were introduced into 0.5 mm of borosilicate capillaries prior to being mounted and aligned on an Empyrean PANalytical powder diffractometer, using Cu Kα radiation (λ = 1.54056 Å). For each sample, five repeated measurements were collected at room temperature (2θ = 2°–60°) and merged in a single diffractogram.

The same procedure was carried out for polycrystalline samples of **3**, **4**, and **5**, after 10 sorption/desorption cycles with the solution containing the five NEOs simultaneously.

## Supporting Information Available

Experimental preparation, analytical characterization; Tables S1–S5, Schemes S1 and S2, and Figures S1–S13 (PDF)
CCDC reference numbers CCDC 2072807-2072809 (CIF), (CIF) and (CIF)

## Supplementary Material

am1c08833_si_001.pdf

am1c08833_si_002.cif

am1c08833_si_003.cif

am1c08833_si_004.cif
