# Supplementary material for: Highly Efficient Removal of Neonicotinoid Insecticides by Thioether-Based (Multivariate) Metal–Organic Frameworks
Source: ACS Appl Mater Interfaces. 2021 Jun 14;13(24):28424–32. doi: 10.1021/acsami.1c08833 (PMC9201812; doi:10.1021/acsami.1c08833)
Supplement: Supplementary file 1 — am1c08833_si_001.pdf [file am1c08833_si_001.pdf]

Supporting Information (SI) for the manuscript:

Highly Efficient Removal of Neonicotinoid Insecticides by Thioether–Based (Multivariate) Metal-Organic Frameworks

Cristina Negro,[†] Hector Martínez Pérez-Cejuela,[‡] Ernesto F. Simó-Alfonso,[‡] Jose Manuel Herrero-Martínez*[,‡], Rosaria Bruno,[§] Donatella Armentano,*[,§] Jesús Ferrando–Soria*[,†] and Emilio Pardo*[,†]

[†]Instituto de Ciencia Molecular (ICMol), Universidad de Valencia, 46980 Paterna, Valencia, Spain. [‡]Departamento de Química Analítica, Universitat de València, c/Dr. Moliner, 50, 46100 Burjassot, Valencia, Spain. [§]Dipartimento di Chimica e Tecnologie Chimiche (CTC), Università della Calabria, Rende 87036, Cosenza, Italy

Experimental Section

Materials. All chemicals were of reagent grade quality. They were purchased from commercial sources and used as received.[1,2] MOFs **1**, **2**, **3** and **4** were prepared as previously reported. Dinuclear precursor complexes $[(Me_4N)_2\{Cu_2[(S,S)$-serimox$](OH)_2\}\cdot 5H_2O$, $[(Me_4N)_2\{Cu_2[(S,S)$-threomox$](OH)_2\}\cdot 4H_2O$, $[(Me_4N)_2\{Cu_2[(S,S)$-methox$](OH)_2\}\cdot 4H_2O$ and $[(Me_4N)_2\{Cu_2[(S,S)$-Mecysmox$](OH)_2\}\cdot 4H_2O$ were prepared as previously reported.[2]

Physical Techniques: Elemental (C, H, S, N), SEM-EDX and ICP-MS analyses were performed at the Microanalytical Service of the Universitat de València. FT–IR spectra were recorded on a Perkin-Elmer 882 spectrophotometer as KBr pellets. The thermogravimetric analyses were performed on crystalline samples under a dry N_2 atmosphere with a Mettler Toledo TGA/STDA 851e thermobalance operating at a heating rate of 10 ºC min^{-1}. Scanning Electron Microscopy coupled with Energy Dispersive X–ray (SEM/EDX) was carried out with a XL 30 ESEM (PHILIPS) microscope equipped with a home–made EDX energy dispersive x–ray detector. The N_2 adsorption-desorption isotherms at 77 K were carried out on crystalline samples of **5**, **acetamiprid@5** and **thiacloprid@5** with a Micromeritics ASAP2020 instrument. Samples were activated at 70 °C under reduced pressure (10^{-6} Torr) for 16 h prior to carry out the sorption measurements.

Analytical experiments:

- Instrumentation:

For the solid-phase extraction (SPE) protocols, manifold (VacElut) with twelve positions (Agilent Technologies, Waldbronn, Germany,) and pump for vacuum N938 Laboport (KNF, Freiburg, Germany) were used. For HPLC conditions, an 1100 series

liquid chromatograph (Agilent Technologies) provided with a quaternary pump, a degasser, a thermostated column compartment, an automatic sampler and equipped with diode-array (DAD) detection. Separation was accomplished with a Kinetex EVO C18 (250 × 4.6 mm, 5 µm particle size, Phenomenex). The mobile phases consisted of water (A) and MeCN (B) delivered at 1 mL min^{-1}. Isocratic elution mode was used to achieve the chromatographic separation (74:26 A:B, v/v) during 15 min. The injection volume was 25 µL, no temperature control was needed and the detection wavelength were fixed at 244 and 270 nm.

X-ray crystallographic data collection and structure refinement: Crystals of **5, acetamiprid@5** and **thiacloprid@5** were selected and mounted on a MITIGEN holder in Paratone oil, then quickly placed in a nitrogen stream cooled at 100 K in order to extract the best data set avoiding the possible degradation upon desolvation or exposure to air. Nevertheless, crystals of both **acetamiprid@5** and **thiacloprid@5** and samples displayed an outstanding stability at air and room temperature for at least four weeks, as demonstrating by their diffraction patterns measured at 296 K as well, without displaying any important crystal decay. Diffraction data for **5** were collected using synchrotron radiation at I19 beamline of the Diamond Light Source at λ = 0.6889 Å, whereas for **acetamiprid@5** and **thiacloprid@5** data were acquired on a Bruker-Nonius X8APEXII CCD area detector diffractometer using graphite-monochromated Mo-K_α radiation (λ = 0.71073 Å), as significant beam-damage was observed for both single crystals under synchrotron radiation. Bearing in mind that crystal structure of adsorbates **acetamiprid@5** and **thiacloprid@5** have been obtained measuring on crystals which suffered a single-crystal to single-crystal (SC to SC) process, it is reasonable. The data were processed through xia2 (**5**),[3] or SAINT[4] reduction and SADABS[5] multi-scan absorption [**acetamiprid@5** and **thiacloprid@5**] software. The

structures were solved with the SHELXS structure solution program, using the Patterson method. The model was refined with version 2018/3 of SHELXL against F^2 on all data by full-matrix least squares.[6]

Indeed, we could measure on **5** at synchrotron, before loading guests, in order to go deep in the point of statistical disorder we observe for the whole series of Multivariate MOFs (MTV-MOFs). In **5, acetamiprid@5** and **thiacloprid@5** crystallographic analysis does give superimposed snapshots of reactant dimers averaged in mixed {Cu^{II}_2[(S,S)-methox/mecysmox]} ones. Despite the highest data quality of data set for **5**, measured up to theta 36° (see Table S1), no appreciable variations in the final best model of crystal structure have been achieved for **5**.

In all samples, all non-hydrogen atoms of the networks were refined anisotropically, except some highly dynamically disordered atoms of guest molecules in **acetamiprid@5** and **thiacloprid@5**. For a such kind of single crystals, the lower data quality for adsorbates, embedding highly disordered guest molecules, makes the use of constraints and especially restraints essential. The use of some bond lengths restraints applied on atoms belonging to highly dynamic moieties, especially during the refinements in **acetamiprid@5** and **thiacloprid@5**, has been reasonably imposed and related to the expected thermal motion, likely depending on the large pore's size of the frameworks (FLAT, DFIX, DANG, SIMU, DELU and ISOR). In particular, aminoacidic chains in all the three samples have been systematically refined with restrains on C-C and C-S bond lengths. In the refinement of **acetamiprid@5** and **thiacloprid@5** crystal structures, some further restrains, to make the refinement more efficient, have been applied. For instance, ADP components have been restrained to be similar to other related atoms, using SIMU 0.04 for disordered sections or EADP for group of atoms of the guest molecules expected to have essentially similar ADPs. In **5**,

all the hydrogen atoms of the networks were set in calculated position and refined isotropically using the riding model. As far as for **acetamiprid@5** and **thiacloprid@5** crystal structure's refinement is concerned, hydrogen atoms on the guest molecules, and for found solvent lattice molecules were neither found nor calculated. Furthermore, while in **acetamiprid@5** they were calculated for the network, in **thiacloprid@5,** due to the impressive statistical disorder, other than dynamical one, where not defined neither for network nor for the guest molecules. In fact, it is often expected that guest molecules are severely disordered, as a direct consequence of their high thermal motion and also exhibited statistic disorder.

As reported in main text as well, the oxamidato-bridged dicopper(II) units of {Cu^{II}_2[(S,S)-mecysmox]} and {Cu^{II}_2[(S,S)-methox]} inserted with a 1:1 ratio in **5** exhibit a statistically disorder in the crystal structure (Figure S1, inset), where the very similar percentage of mecysmox and methox leads to a completely superimposed snapshot of mixed {Cu^{II}_2[(S,S)-methox/mecysmox]} dimers, synchrotron measurements on **5** sample unveiled the same issue for its crystal structure. In fact, such disorder gives a mixed view of **5**, understandable considering that a crystal structure is the *spatial average*, of all molecules/fragments, together with all their possible orientations averaged, in the crystal *via* only one unit cell. It should be also underlined that our best model has taken into account the most persistence conformations –within the additional complication– that even a low percentage, not taken into account, such as a 10% of a whole pesticides guest molecule, is still a significant amount of electron density, and whole-molecule disorder lurks everywhere in the inky shadows of structure refinement, affecting quality of the model.

Both in **acetamiprid@5** and **thiacloprid@5** the occupancies of the guests in the pores, have been defined by ICP-MS analyses and, in the crystal structure, fixed at

0.333. We strongly believe that it is the more reliable way to accurately define loading instead of taking into account merely thermal factors, which can be affected by a lot of issues above all severe disorder.

In **acetamiprid@5**, acetamiprid guest molecules are statistically disordered exhibiting three set of possible orientations of the guest molecules as detailed in Figure S8 and S12. Similarly, in **thiacloprid@5,** thiacloprid pollutants are severely thermally and statistically disordered (Figure S10 and S13), which is probably related to the different degrees of freedom, related to diverse possible conformations. This fact imposed to block atoms N3L, N4L, C11L and Cl1 terminal groups during the final refinement. In particular, the partial overlap between different orientations at atom sites, in both adsorbate structures, make them disordered with carbon and nitrogen sites (see Figures S8, S10, S12 and S13).

The contribution to the diffraction pattern from the highly disordered solvent molecules in **5**, located in the voids, was subtracted from the observed data through the SQUEEZE method, implemented in PLATON.[7]

Finally, the estimated empty volumes for **5, thiacloprid@5** and **acetamiprid@5** without the crystallization water molecules is 1233.2 (1), 829.6 (2) and 379.7 (3) Å^3, values which represent *ca.* 34.6, 22.6 and 10.3 %, respectively, of potential void per unit cell volume [V = 3562.68(3), 3672.6 (2) and 3679.5 (3) Å^3]. In accordance with BET and SCXRD analysis, the channels of **5** are entirely filled by organic molecular guests with the help of solvent molecules.

A summary of the crystallographic data and structure refinement for the three compounds is given in Table S2. The comments for the alerts A and B are reported in the CIFs using the validation response form (vrf). CCDC reference numbers are 2072807-2072809 for **5**, **acetamiprid@5** and **thiacloprid@5**, respectively.

The final geometrical calculations on free voids and the graphical manipulations were carried out with PLATON[7] implemented in WinGX,[8] and CRYSTAL MAKER[9] programs, respectively.

Table S1. Molecular structures of NEOs and their water/octanol partition coefficients.

Name	Structure	Log P
Acetamiprid	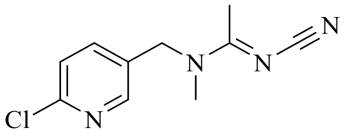	0.80
Thiacloprid	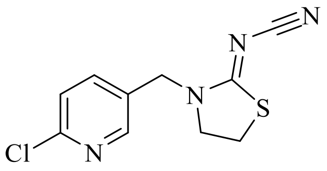	1.26
Imidacloprid	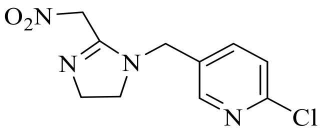	0.57
Clothianidin	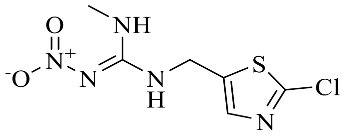	0.70
Thiamethoxan	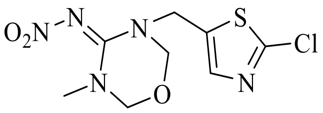	-0.13

Table S2. Summary of Crystallographic Data for **5**, **acetamiprid@5** and **thiacloprid@5**

Compound	**5**	**acetamiprid@5**	**thiacloprid@5**
Formula	$C_{33}H_{118}SrCu_6N_6O_{57}S_6$	$C_{43}H_{75}SrCu_6ClN_{10}O_{30}S_6$	$C_{43}H_{91}SrCu_6ClN_{10}O_{39}S_7$
M (g mol^{-1})	2172.55	1908.80	2100.98
λ (Å)	0.6889	0.71073	0.71073
Crystal system	hexagonal	hexagonal	hexagonal
Space group	$P6_3$	$P6_3$	$P6_3$
a (Å)	17.82070(5)	18.0282(8)	18.0090(8)
c (Å)	12.95380(6)	13.0723(8)	13.0756(8)
V (Å^3)	3562.68(2)	3679.5(4)	3672.6(4)
Z	2	2	2
ρ_{calc} (g cm^{-3})	2.025	1.723	1.900
μ (mm^{-1})	2.478	2.710	2.761
T (K)	100	100	100
θ range for data collection (°)	1.989 to 36.049	2.032 to 26.224	2.262 to 26.161
Completeness to θ = 25.0	100%	100%	100%
Measured reflections	78315	50563	43839
Unique reflections (Rint)	11771 (0.0433)	4941 (0.0569)	4918 (0.0567)
Observed reflections [$I > 2\sigma(I)$]	6217	3812	3629
Goof	0.981	1.075	1.575
Absolute structure parameter (Flack)	0.39(2)	0.44(2)	0.41(2)
R^a [$I > 2\sigma(I)$] (all data)	0.0574 (0.0929)	0.0835 (0.1060)	0.0693 (0.0949)
wR^b [$I > 2\sigma(I)$] (all data)	0.1847 (0.1718)	0.2488 (0.2761)	0.2149 (0.2274)
CCDC	2072807	2072808	2072809

^{a} $R = \sum(|F_o| - |F_c|)/\sum|F_o|$. ^{b} $wR = [\sum w(|F_o| - |F_c|)^2/\sum w|F_o|^2]^{1/2}$.

Table S3. Removal values for NEOs from 1 mg L^{-1} of aqueous samples using different BioMOFs (n=3).

**Neonicotinoids**	**Removal of different BioMOFs (%)**				
	**MOF-1**	**MOF-2**	**MOF-3**	**MOF-4**	**MOF-5**
**Thiamethoxam**	2	11	20	10	36
**Clothianidin**	16	11	32	33	69
**Imidacloprid**	17	12	37	28	68
**Acetamiprid**	17	11	69	90	98
**Thiacloprid**	22	14	90	96	99

Experimental conditions: loading 1 mL of aqueous standards with 1 mg L^{-1} of each contaminant, washing 1mL of water and elution 5 mL of MeOH. The removal was quantified, after filtrating with nylon membrane 0.22 µm, by HPLC-UV.

Table S4. NEOs removal efficiencies[1] from river water using **MOF-3-5** (n=3).

**Neonicotinoids**	**Removal of different BioMOFs (%)**		
	**MOF-3**	**MOF-4**	**MOF-5**
**Thiamethoxam**	21	34	58
**Clothianidin**	45	42	77
**Imidacloprid**	40	42	78
**Acetamiprid**	96	77	100
**Thiacloprid**	99	95	100

[1]Data obtained from Figure S6.

Experimental conditions: loading 1 mL of river water spiked with 5 mg L^{-1} of each contaminant, washing 1mL of water and elution 5 mL of MeOH. The removal was quantified, after filtrating with nylon membrane 0.22 µm, by HPLC-UV.

Table S5. Precision shown as maximum relative standard deviation of different BioMOFs as SPE sorbents in the analysis of NEOs.

Compound	Precision, RSD (n=3)	
	**Intra-device[a]**	**Inter-device**
Thiamethoxam	2.3; 5.3	7.9
Clothianidin	4.0; 7.7	8.0
Imidacloprid	3.5; 7.5	8.6
Acetamiprid	4.2; 6.0	7.2
Thiacloprid	2.1; 3.9	6.7

[a]As intra- and inter-day RSD (n = 3)
The procedure was carried out using 1 mL of standard mixture (1 mg L^{-1}) of each NEO and the removal was quantified, after filtrating with nylon membrane 0.22 µm, by HPLC-UV.

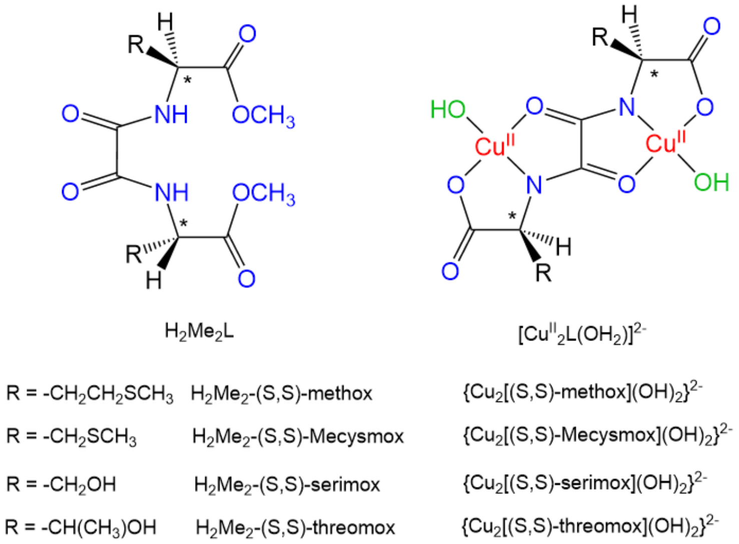

Scheme S1. Chemical structures of the chiral bis(amino acid)oxalamide ligands (left), highlighting the potential coordination sites and chiral centers (*) and the corresponding dianionic bis(hydroxo) dicopper(II) complexes (right).

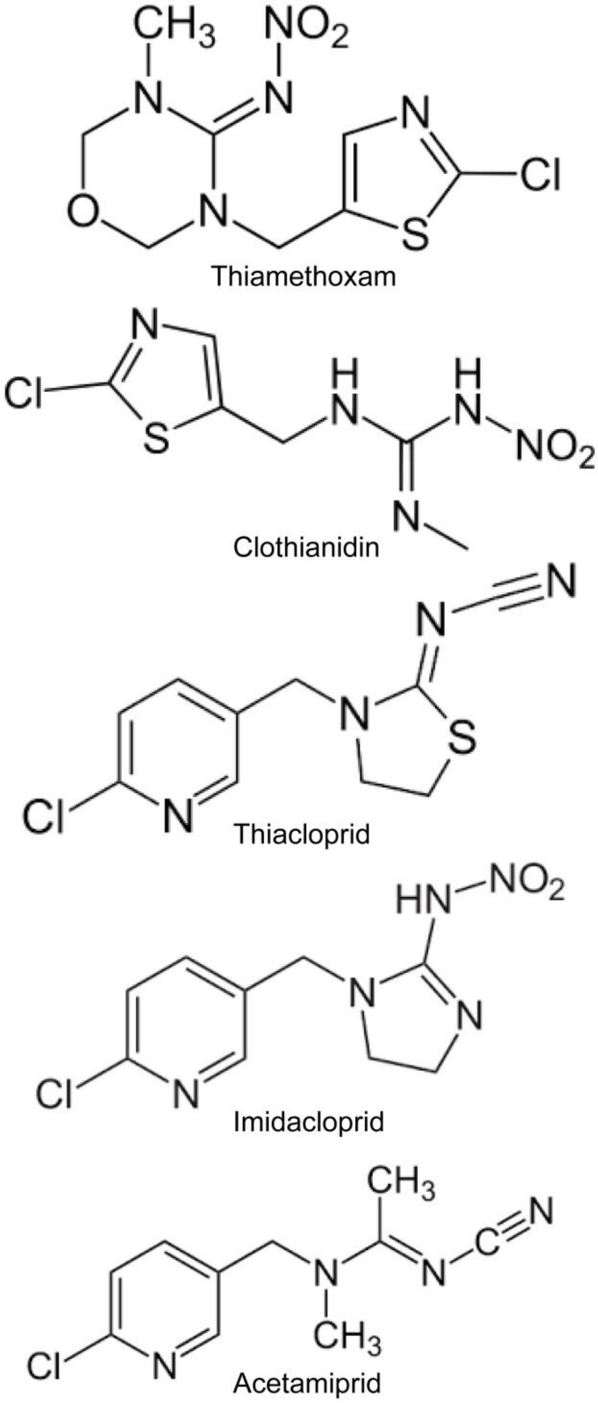

Scheme S2. Chemical structures of the neonicotinoids thiacloprid, acetamiprid, clothianidin, imidacloprid and thiamethoxam.

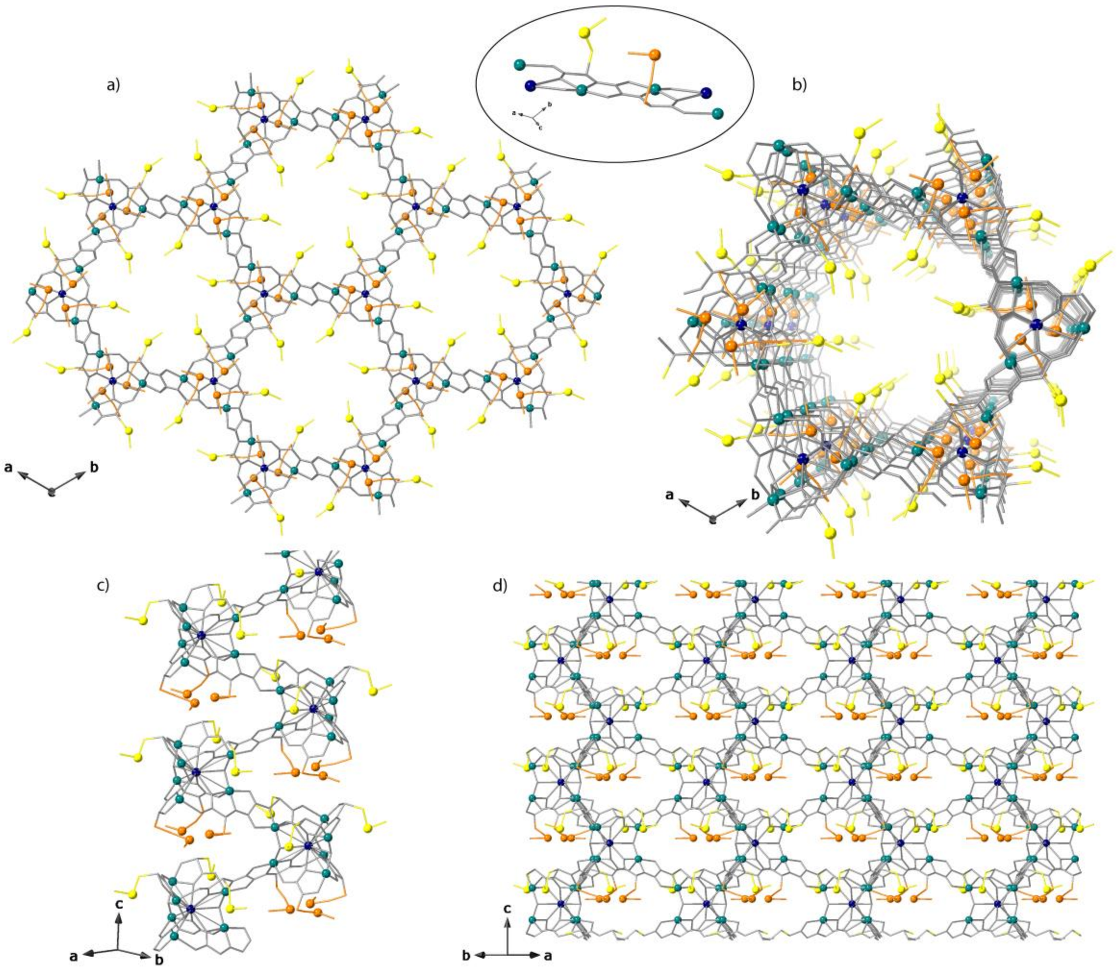

Figure S1. View down *c* axis of the porous structure of $\{Sr^{II}Cu^{II}_6[(S,S)$- methox$]_{1.50}[(S,S)$-Mecysmox$]_{1.50}$ $(OH)_2(H_2O)\}\cdot 36H_2O$ **5**. (a) Perspective view along *c* crystallographic axis of a single channel of the MTV–MOF **5**. (b) Views along [111] direction of (c) the chiral rods and (d) a portion of the porous crystal structure of MTV–MOF **5**. The crystallization water molecules are omitted for clarity. The inset shows the superimposed snapshot of mixed $\{Cu^{II}_2[(S,S)$-methox/mecysmox$]\}$ dimers, on which crystallographic model of **5** is based. Organic ligands are depicted as gray sticks, whereas the amino acid residues are represented with the following color code: $–CH_2SCH_3$ (yellow) and $–CH_2CH_2SCH_3$ (orange). Strontium, copper, and sulfur atoms are shown as blue, cyan, and yellow (for methylcysteine fragment)/orange (for methionine fragment) spheres, respectively.

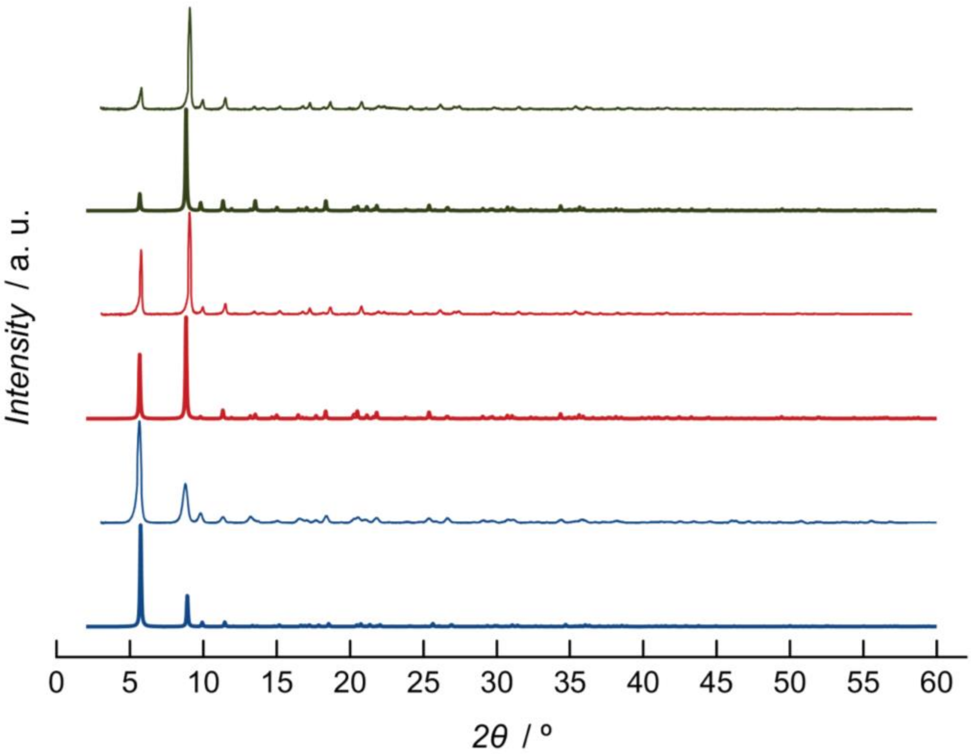

Figure S2. Theoretical (bottom) and experimental (top) PXRD patterns of **5** (blue), **acetamiprid@5** (red) and **thiacloprid@5** (green) in the 2.0–60.0°.

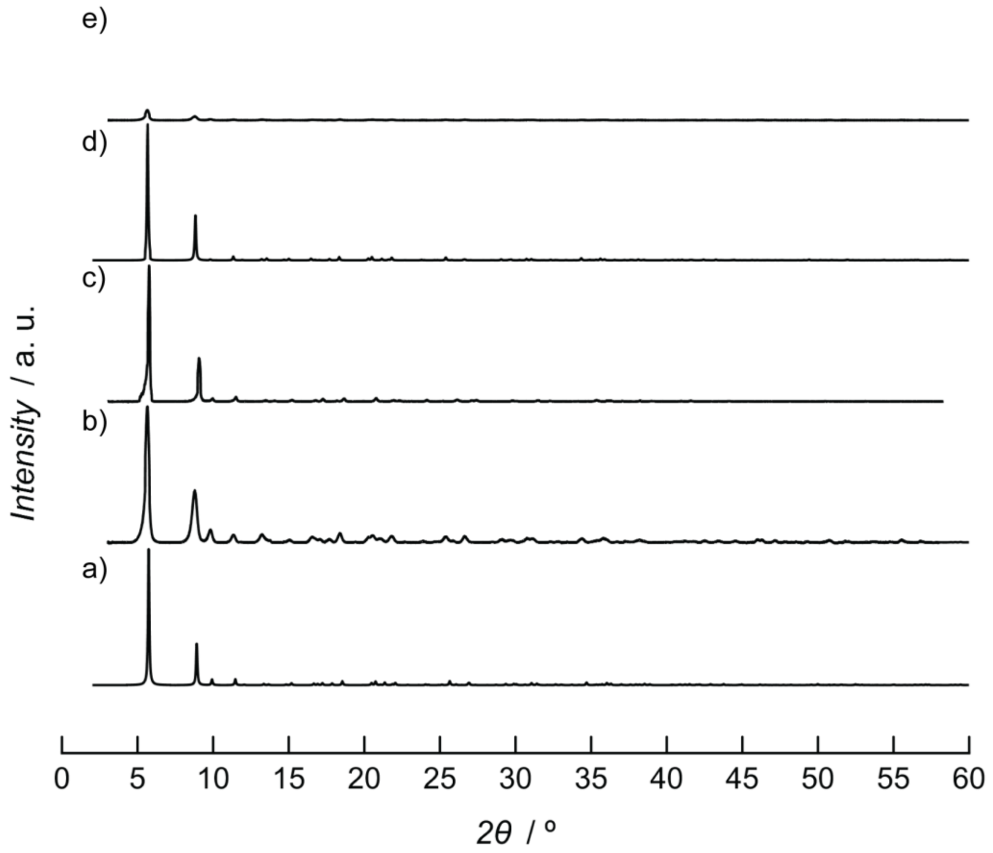

Figure S3. (a), Calculated PXRD pattern profile of **5**. PXRD pattern profiles of **5** after 48 h immersed in pH = 7 (b), pH = 12 (c), pH = 5 (d) and pH = 2 (e) aqueous solutions in the 2.0–60.0°.

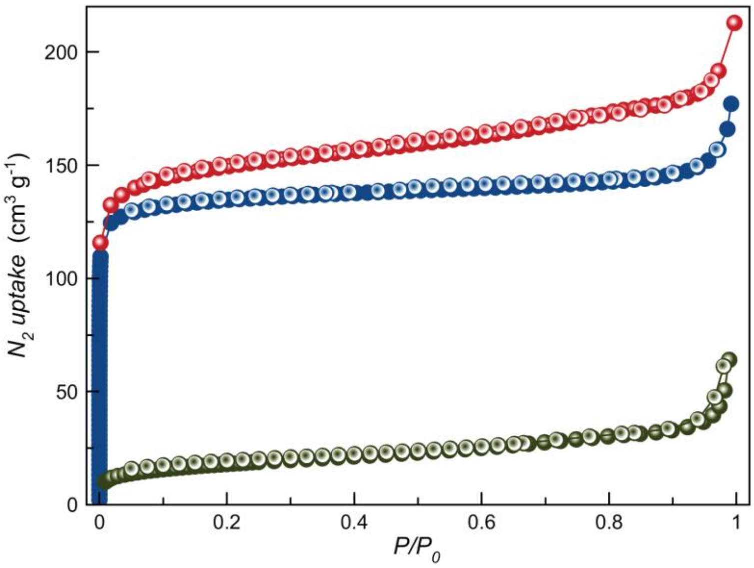

Figure S4. N_2 (77 K) adsorption isotherms for the activated compounds **3** (green), **4** (red) and **5** (blue). Filled and empty symbols indicate the adsorption and desorption isotherms, respectively. The samples were activated at 70 °C under reduced pressure for 16 h prior to carry out the sorption measurements.

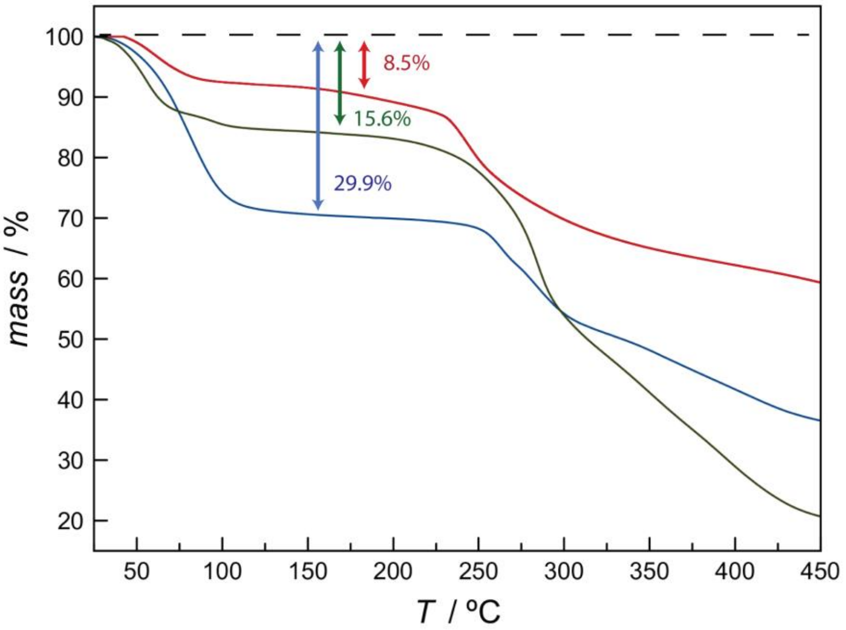

Figure S5. Thermo-Gravimetric Analysis (TGA) of **5** (blue), **acetamiprid@5** (red) and **thiacloprid@5** (green) under dry N_2 atmosphere.

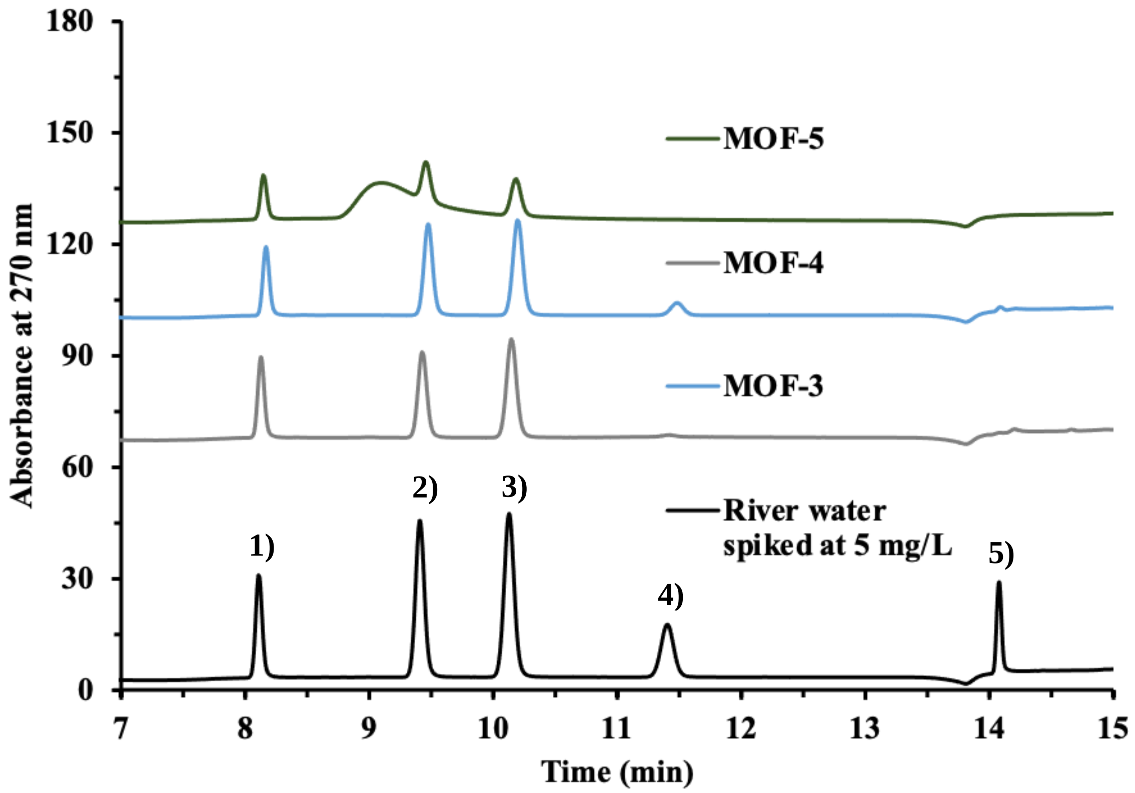

Figure S6. HPLC-DAD chromatograms from a river water spiking at 5 mg L^{-1} of each analyte without SPE treatment (black line) and with SPE treatment (grey, blue and green lines). Chromatographic details are given in the Experimental and Supplementary Section. Peak identification: 1) thiamethoxan; 2) clothianidin; 3) imidacloprid; 4) acetamiprid and 5) thiacloprid.

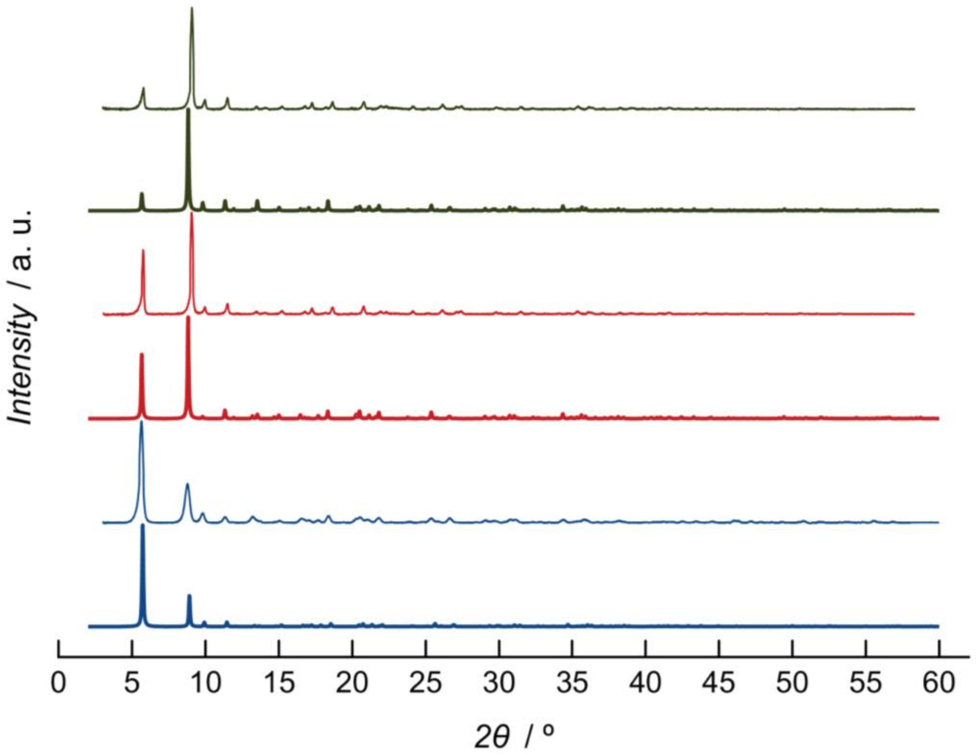

Figure S7. Theoretical (bottom) and experimental (top) PXRD patterns of **3** (blue), **4** (red) and **5** (green), after 10 sorption-desorption cycles in the 2.0–60.0° range.

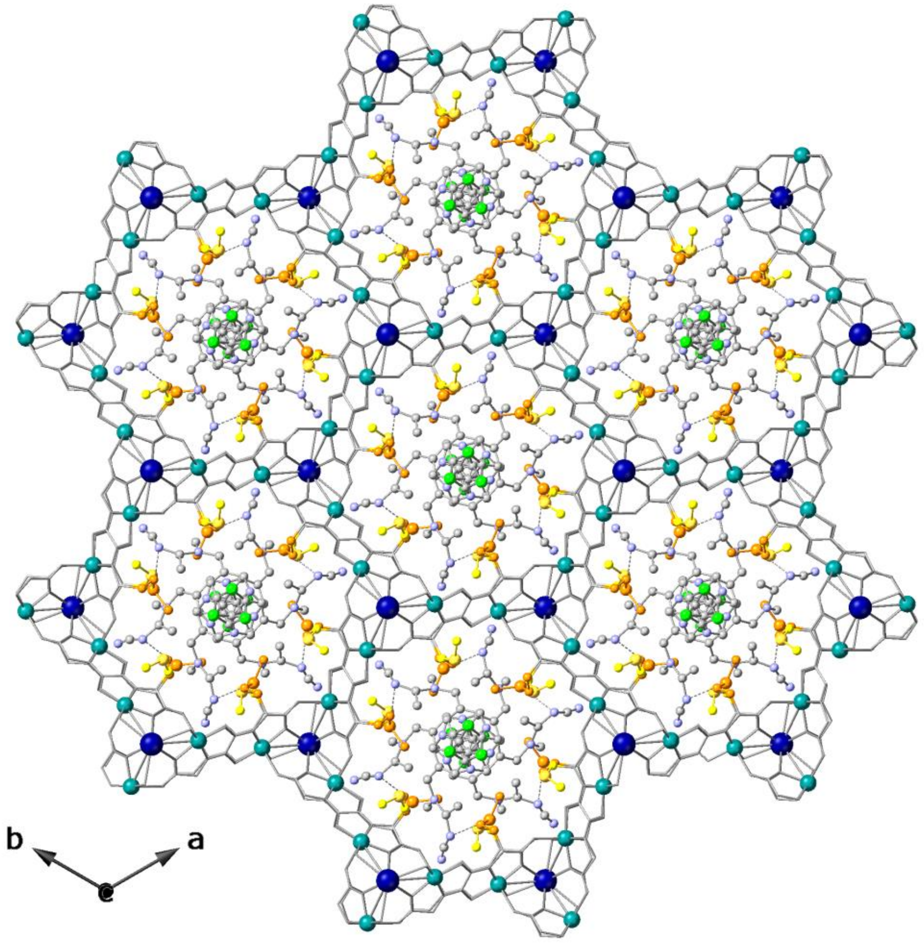

Figure S8. View of the crystal structure of **acetamiprid@5** along the *c* axis (the crystallization water molecules are omitted for clarity) undoubtedly showing pores filled by acetamiprid molecules as guests. Organic ligands of the network are depicted as gray sticks, whereas the amino acid residues are represented with the following color code: –CH_2SCH_3 (yellow) and –$CH_2CH_2SCH_3$ (orange). Guest molecules are depicted as balls and sticks with carbon grey, nitrogen light blue and chlorine green. Strontium, copper, and sulfur atoms are shown as blue, cyan, and yellow (for methylcysteine fragment)/orange (for methionine fragment) spheres, respectively.

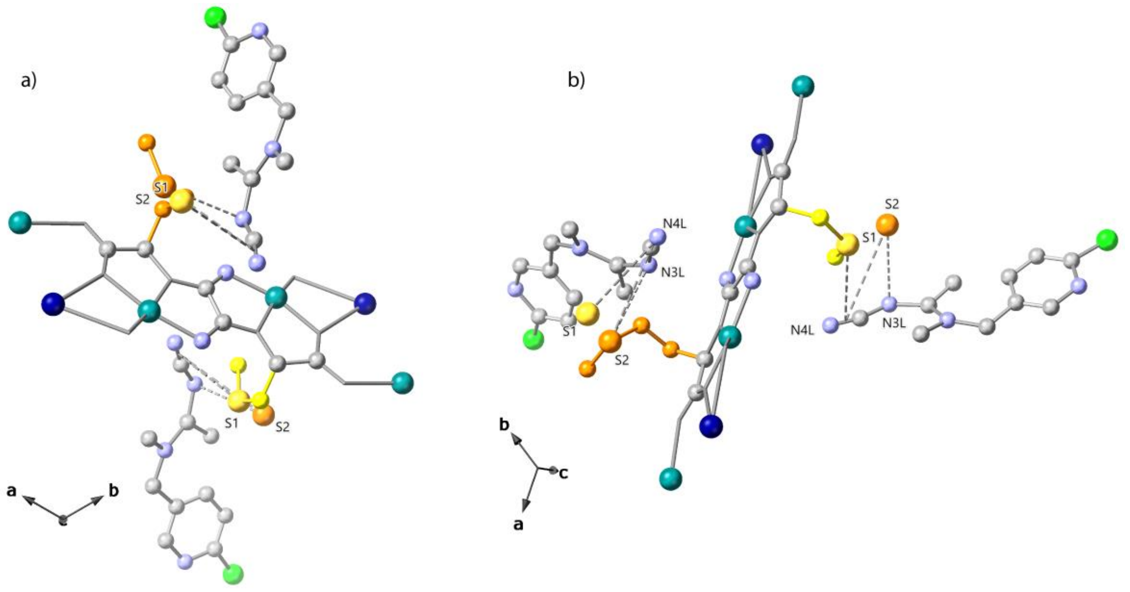

Figure S9. Details of host-guest interactions along *c* axis (a) and channel's propagation direction (b) in **acetamiprid@5**, unveiled by single crystal X-ray analysis. The S···N-CN involving only methionine residues [S···N distances of 3.18(1) Å] and S···nitrile interactions, involving both kind of *arms* blocking acetamiprid terminal moieties [S···$CN_{Centroid}$ distances of 3.67(1) and 3.82(1) Å, for methyl-cysteine and methionine residues, respectively] are depicted with dashed lines. Organic ligands of the network and guest molecules are depicted as ball and sticks with carbon grey, nitrogen light blue and chlorine green. Strontium, copper and sulfur atoms are shown as blue, cyan and yellow (for methylcysteine fragment)/orange (for methionine fragment) spheres, respectively. The amino acid residues are represented with the following color code: $–CH_2SCH_3$ (yellow) and $–CH_2CH_2SCH_3$ (orange).

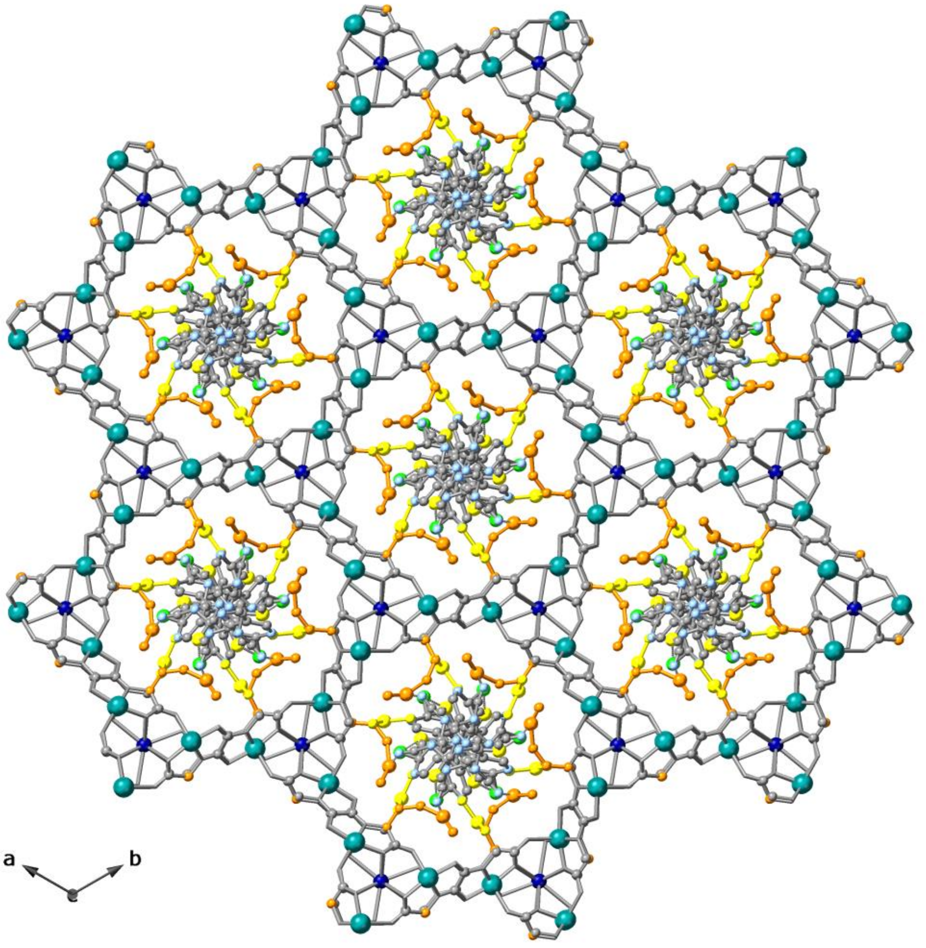

Figure S10. View of the crystal structure of **thiacloprid@5** along the *c* axis (the crystallization water molecules are omitted for clarity) markedly showing pores filled by thiacloprid molecules as guests. Organic ligands of the network are depicted as gray sticks whereas the amino acid residues are represented with the following color code: $–CH_2SCH_3$ (yellow) and $–CH_2CH_2SCH_3$ (orange). Guest molecules are depicted as balls and sticks with carbon grey, nitrogen light blue and chlorine green. Strontium, copper, and sulfur atoms are shown as blue, cyan and yellow (for methylcysteine fragment)/orange (for methionine fragment) spheres, respectively.

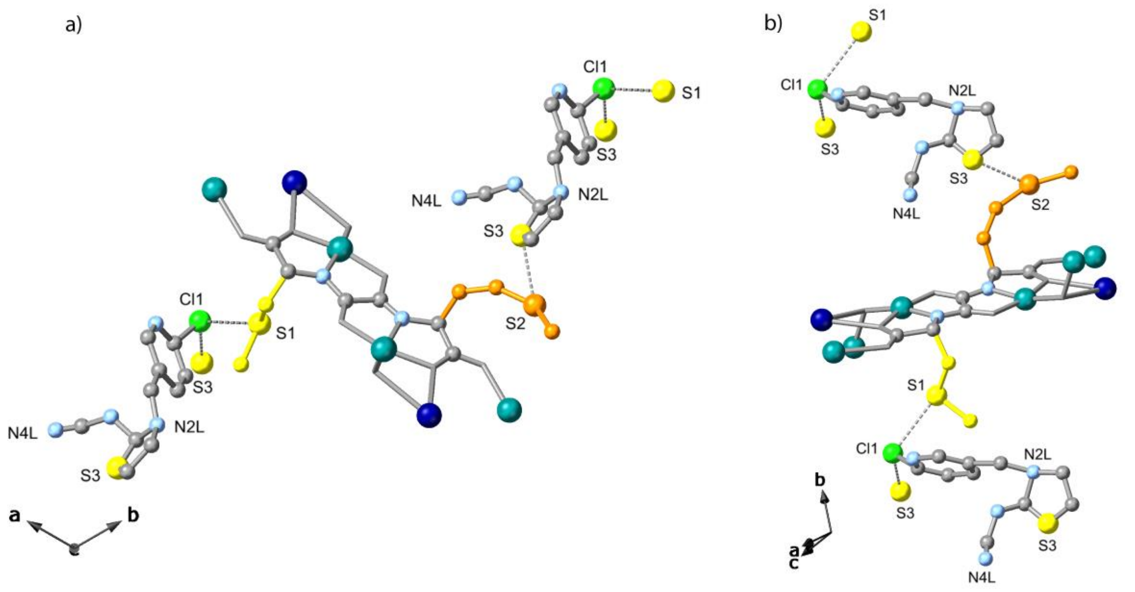

Figure S11. Details of host-guest interactions in **thiacloprid@5**, unveiled by single crystal X-ray analysis. The S···Cl interaction [distance of 3.10(1) Å] and the interactions of the type S···S held with thiazolidine ring of pollutants molecules at a S···S distance of 2.76(1) Å, are depicted with dashed lines. Strontium, copper, and sulfur atoms are shown as blue, cyan and yellow (for methylcysteine fragment)/orange (for methionine fragment) spheres, respectively.

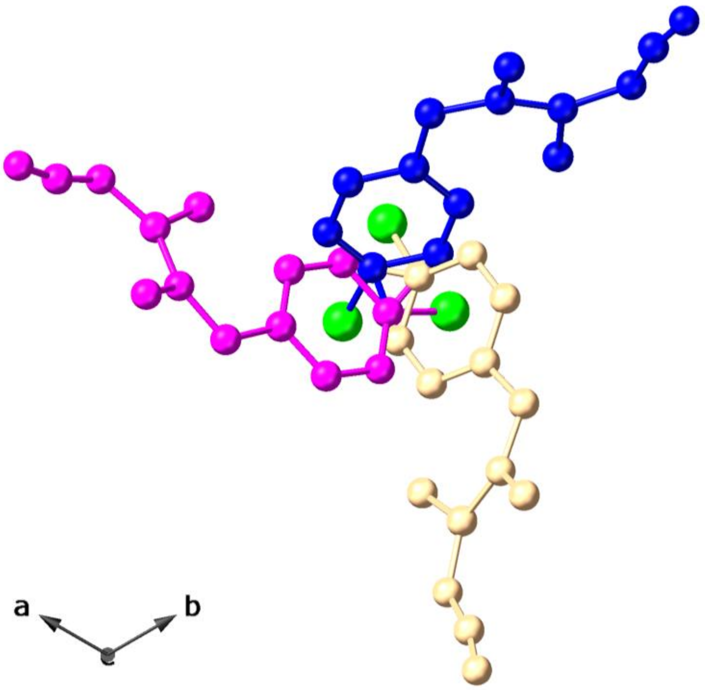

Figure S12. Details of disordered acetamiprid guest's molecule. The three sets of different conformations allowed by $P6_3$ space group, are depicted in purple, blue and amber, respectively.

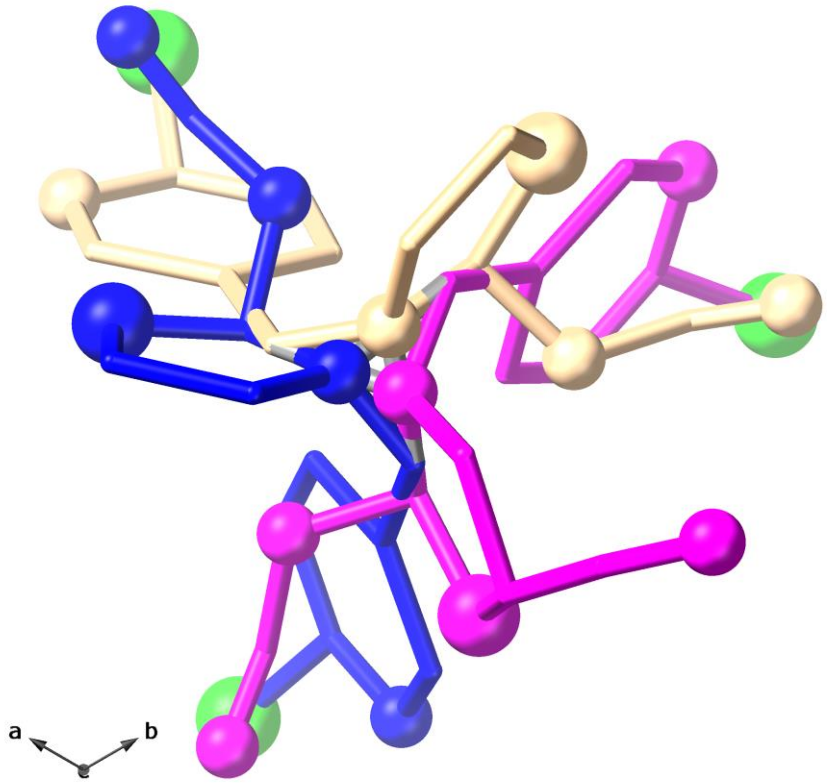

Figure S13. Details of disordered thiacloprid guest's molecules. The three sets of different conformations allowed by $P6_3$ space group, are depicted in purple, blue and amber, respectively.

References

(1) (a) M. Mon, R. Bruno, J. Ferrando-Soria, L. Bartella, L. Di Donna, M. Talia, R. Lappano, M. Maggiolini, D. Armentano, E. Pardo, *Mater. Horizons* **2018**, *5*, 683–690. (b) M. Mon, R. Bruno, R. Elliani, A. Tagarelli, X. Qu, S. Chen, J. Ferrando-Soria, D. Armentano, E. Pardo, *Inorg. Chem.* **2018**, *57*, 13895–13900.

(2) (a) E. Tiburcio, R. Greco, M. Mon, J. Ballesteros-Soberanas, J. Ferrando-Soria, M. López-Haro, J. C. Hernández-Garrido, J. Oliver-Meseguer, C. Marini, M. Boronat, et al., *J. Am. Chem. Soc.* **2021**, *143*, 2581–2592. (b) H. M. Pérez-Cejuela, M. Mon, J. Ferrando-Soria, E. Pardo, D. Armentano, E. F. Simó-Alfonso, J. M. Herrero-Martínez, *Microchim. Acta* **2020**, *187*, 201. (c) M. Mon, J. Ferrando-Soria, T. Grancha, F. R. Fortea-Pérez, J. Gascon, A. Leyva-Pérez, D. Armentano, E. Pardo, *J. Am. Chem. Soc.* **2016**, *138*, 7864–7867. (d) M. Mon, F. Lloret, J. Ferrando-Soria, C. Martí-Gastaldo, D. Armentano, E. Pardo, *Angew. Chemie Int. Ed.* **2016**, *55*, 11167–11172.

(3) (a) Evans, P. Sclaing and assessment of data quality. *Acta Cryst. D* **62**, 72–82 (2006). (b) Evans, P. R., Murshudov, G. N. How good are my data and what is the resolution?. *Acta Cryst. D* **69**, 1204–1214 (2013). (c) Winn, M. D.; Ballard, C. C.; Cowtan, K. D.; Dodson, E. J.; Emsley, P.; Evans, P. R.; Keegan, R. M.; Krissinel, E. B.; Leslie, A. G. W.; McCoy, A.; McNicholas, S. J.; Murshudov, G. N.; Pannu, N. S.; Potterton, E. A.; Powell, H. R.; Read, R. J.; Vagin, A.; Wilsonc K. S. Overview of the *CCP4* suite and current developments. *Acta Cryst. D* **67**, 235–242 (2011). (d) Winter, G. *xia2*: and expert system for macromolecular crystallography data reduction. *J. Appl. Cryst.* **43**, 186–190 (2010). (e) Winter, G.; Waterman, D. G.; Parkhurst, J. M.; Brewster, A. S.; Gildea, R. J.; Gerstel, M.; Fuentes-Montero, L.; Vollmar, M.; Michels-Clark, T.; Young, I. D.; Sauterb, N. K.; Evansa, G. *DIALS*: implementation and evaluation of a new integration package *Acta Cryst*. **2018**, D74, 85-97

(4) SAINT, version 6.45, Bruker Analytical X-ray Systems, Madison, WI, 2003.

(5) Sheldrick G.M. SADABS Program for Absorption Correction, version 2.10, Analytical X-ray Systems, Madison, WI, 2003.

(6) (a) Sheldrick, G. M. Crystal structure refinement with SHELXL. *Acta Cryst. C* **71**, 3–8 (2015). (b) Sheldrick, G. M. A short history of SHELX. *Acta Cryst. A* **64**, 112–122 (2008). (c) SHELXTL-2013/4, Bruker Analytical X-ray Instruments, Madison, WI, 2013.

(7) (a) Spek, A. L. *PLATON* SQUEEZE: a tool for the calculation of the disordered solvent contribution to the calculated structure factors. *Acta Crystallogr. Sect. C-Struct. Chem*. **71**, 9–18 (2015). (b) Spek, A. L. Structure validation in chemical crystallography. *Acta Crystallogr. Sect. D, Biol. Crystallogr.* **65**, 148–155 (2009).

(8) Farrugia, L. J. *WinGX* suite for small-molecule single-crystal crystallography. *J. Appl. Crystallogr.* **32**, 837–838 (1999).

(9) Palmer, D. CRYSTAL MAKER, Cambridge University Technical Services, C. No Title, 1996.
